# Inhibitory Copulation Effect of Vibrational Rival Female Signals of Three Stink Bug Species as a Tool for Mating Disruption

**DOI:** 10.3390/insects12020177

**Published:** 2021-02-18

**Authors:** Aline Moreira Dias, Miguel Borges, Maria Carolina Blassioli Moraes, Matheus Lorran Figueira Coelho, Andrej Čokl, Raúl Alberto Laumann

**Affiliations:** 1 Zoology Post-Graduation Program, Institute of Biology, University of Brasilia, Brasília 70910-900, Brazil; linem.dias@gmail.com; 2 Semiochemicals Laboratory, Embrapa Genetic Resources and Biotechnology, Brasília 70770-917, Brazil; miguel.borges@embrapa.br (M.B.); carolina.blassioli@embrapa.br (M.C.B.M.); matheuslorran8856@gmail.com (M.L.F.C.); 3 Department of Organisms and Ecosystems Research, National Institute of Biology, Ljubljana 1000, Slovenia; andrej.cokl@nib.si

**Keywords:** biotremology, behavior manipulation, population control, pest management

## Abstract

**Simple Summary:**

In this work, we investigated the effects of conspecific female rival signals in vibratory communication and mating behavior of three species of stink bugs. In the presence of rival female signals, as noisy background vibrations, couples (a male and a female) of the three species showed negative effects in their sexual vibratory communication that resulted in reduced mating and copulation in relation to pairs not exposed to rival signals. The results suggest that female rival signals could be used to disrupt mating and may be a tool for stink bug management by reducing their population increase.

**Abstract:**

Stink bugs are major pests in diverse crops around the world. Pest management strategies based on insect behavioral manipulation could help to develop biorational management strategies of stink bugs. Insect mating disruption using vibratory signals is an approach with high potential for pest management. The objective of this work was to investigate the effect of conspecific female rival signals on the mating behavior and copulation of three stink bug species to establish their potential for mating disruption. Previously recorded female rival signals were played back to bean plants where pairs of the Neotropical brown stink bug, *Euschistus heros*, and two green stink bugs, *Chinavia ubica* and *Chinavia impicticornis* were placed. Vibratory communication and mating behavior were recorded for each pair throughout the experimental time (20 min). Female rival signals show a disrupting effect on the reproductive behavior of three conspecific investigated stink bug species. This effect was more clearly expressed in *E. heros* and *C. ubica* than in *C. impicticornis*. The likelihood of copulating in pairs placed on control plants, without rival signals, increased 29.41 times in *E. heros*, 4.6 times in *C. ubica* and 1.71 times in *C. impicticornis*. However, in the last case, the effect of female rivalry signals in copulation was not significant. The effect of mating disruption of female rival signals of the three stink bug species may originate from the observed reduction in specific vibratory communication signals emitted, which influences the duet formation and further development of different phases of mating behavior. Our results suggest that female rival signals have potential for application in manipulation and disruption of mating behavior of stink bugs. Further work needs to focus on the effects of female rival signals used in long duration experiments and also their interactions with chemical communication of stink bugs.

## 1. Introduction

Communication using substrate-borne vibrations is common in many insect species, particularly those that live on plants [1]. Among other tasks, substrate-borne vibratory signals enable mate recognition and location on continuous substrate [2]. 

Plant-dwelling stink bugs communicate during reproductive behavior predominantly by chemical [3] and substrate-borne vibratory signals [2]. In these insects, the male pheromone attracts females [4] to land on the same plant, and there it triggers the female to produce vibratory signals that attract males to search for and approach the calling female. Duetting with calling-song signals changes at close distance to mutual emission of the courtship song. Close-range mechanical and visual interactions are the complementary source of information that leads to copulation [2]. In general, stink bug vibratory signals are classified by their specific function in the mating behavioral context, as calling, courtship, copulatory, repelling and rival songs [5]. Their species and gender specificity are expressed by temporal (duration, repetition time) and spectral (dominant frequency, amplitude (AM) and frequency (FM) modulation) characteristics of pulses and pulse trains [2]. The basic repertory of stink bug vibratory signals is produced by vibration of abdomen [2]. Signals produced by tremulation of the whole body, percussion and vibration of lifted wings have been described in the Neotropical brown stink bug, *Euschistus heros* (Fabricius, 1798) [6]; the role of these signals has not been described yet.

Male rival signals have been described in several stink bug species when a group of males competed for access to copulation with the same female [5,7,8,9]. Rivalry between females has been described among Pentatomidae in *E. heros*, two green Neotropical stink bugs *Chinavia ubica* (Rolston, 1983) and *Chinavia impicticornis* (Stål, 1872) [10] and in the southern green stink bug, *Nezara viridula* (L., 1758) [11]. 

Male rivalry interactions are usually characterized by a sequence of alternated short pulses between the competing males. In general, the duration of male rivalry interactions is short (several seconds) and it ends when one of the males is silenced by the competitor [5]. 

Rivalry in females starts by the exchange of calling song signals. In *E. heros* and *C. ubica,* the exchange of these signals stimulates the emission of a specific rival song by one of the competing females that silences the other in *C. ubica* but not in *E. heros*. On the other hand, rivalry in *C. impicticornis* is expressed by the evolution of synchronized exchange of the first type calling song pulse trains (FS-1a) to the emission by a female of the second type of calling song (FS-1b) or to a sequence of readily repeated single pulses that silence the other female [10]. Rivalry interactions are complemented, in some cases, in both investigated *Chinavia* species with physical aggression between individuals [10]. More complex rival interactions have been described for *N. viridula* [12]. In this species, the presence of a male in a group of females triggers rival interactions that start with female calling songs alternation with pulses that occasionally overlap each other. In this phase of interaction, the leader female maintains more or less stable the temporal and spectral characteristics of their pulses and the other tries to disturb changing signal parameters. After this phase, rival interactions evolve to emission of specific rival songs. Three different types of rival songs could be identified [11].

Rival interactions between females appear to have an inhibitory or interference effect on *Chinavia* spp. copulation. In groups with females competing for a male, a reduced number of copulations were observed [10] in relation to those observed in single pairs [12]. However, in *E. heros*, the observed reduction was not of the same magnitude as in *Chinavia* spp. [10]. The principal interference in the reproductive behavior observed during rival female interaction appears to be due the rival signals, but it was not directly tested [10]. 

Biotremology offers great potential for application in pest management by methods and technology with low environmental impact. Vibratory signals could be used to manipulate the behavior of insects, for their monitoring and control [13,14,15,16]. Mating disruption using vibratory signals is one of the proposed approaches [17,18]. The potential use of background noise as mating disruption elements has been shown in several hemipterans, such as the leafhoppers (Cicadellidae) *Amrasca devastans* (Dist.) [19], *Scaphoideus titanus* Ball [17,18,20], *Homalodis cavitripennis* (Germar) [21], the planthopper (Delphacidae), *Nilaparvata lugens*(Stål) [19] and the psyllids *Bactericera cockerelli* ((Šulc, 1909) (Triozidae) [22] and *Diaphorin acitri* (Kuwayama, 1908) (Liviidae) [23,24]. The disrupting effect of pure tone vibrations on the reproductive behavior of *E. heros* was studied [25]. Pure tone vibrations between 75 and 200 Hz significantly reduced the proportion of males searching for females and consequently inhibited copulation. However, in 24 h long experiments, background noise delayed but did not completely disrupt mating [25]. 

Stink bugs are pests in different crops, including legumes, grains, vegetables, fruits and nuts [26]. Their polyphagous and wide geographic distribution makes many stink bug species key pests around the world [27]. In addition, some tropical and subtropical stink bug species, such as *Halyomorpha halys* (Stål), *Bagrada hilaris* (Burmeister) or *Piezodorus guildinii* (Westwood), are invasive in temperate regions, with the potential to become important pests in many crops [28]. In Brazil, stink bugs are the main pests in soybean and other crops, where they usually appear as a complex of species dominated by *E. heros* [29,30]. *E. heros* is a Neotropical stink bug, present principally in tropical and subtropical regions, from Central America to the north of Argentina and Uruguay [26]. *Chinavia ubica* and *Chinavia impicticornis* are considered secondary pests in soybean and are usually found in low densities in soybean fields [30]. These species have similar geographic distribution that includes tropical and subtropical regions in South America, principally Brazil [26]. Management of stink bugs is conducted principally by population monitoring and insecticide applications [31]. The development of biorational control methods based on the manipulation of stink bug behavior could contribute to developing technology with a low environmental impact on agriculture [32]. In this work, the effect of conspecific female rival signals, as background environmental noise, on the mating behavior and copulation of three stink bug species, *E. heros*, *C. ubica* and *C. impicticornis*, was investigated for the first time. The principal objective was to identify the potential of rival signals to be used in a mating disruption strategy for these pests. Two hypotheses were tested by playback experiments: 1—playback of rival female signals inhibits the vibratory communication of conspecific stink bugs; 2—the presence of rival signals reduces the proportion of pairs developing mating behavior and copulation. 

The model species were selected because they have different female rival interactions and signals that could condition the responses of the insects. In addition, the three species give the opportunity to work with a key pest of grain crops in Brazil, *E. heros*, and two secondary pests, *C. ubica* and *C. impicticornis.* In this way, results of this work could help to develop biorational management strategies for current and potential pests.

## 2. Material and Methods

### 2.1. Insects

The colonies of *E. heros*, *C. ubica* and *C. impicticornis* were started with insects collected in soybean fields near Brasília, DF, Brazil. Colonies were maintained for more than 5 years and 60 generations in the laboratory and were partially renewed every year by the incorporation of new field collected insects. Stink bugs were reared following procedures previously described by Borges et al. [33] for *E. heros* and Blassioli-Moraes et al. [34] for *Chinavia* species. Insects were maintained in rearing rooms at 26 ± 10 °C, 65 ± 10% RH, photoperiod 14 hL:10 hD, at the Laboratório de Semioquímicos of EmbrapaRecursosGenéticos e Biotecnologia. Adults were maintained in 8 L transparent plastic containers on a diet composed of green bean pods (*Phaseolus vulgaris* L.), dry soybean seeds (*Glycine max* L.), sunflower seeds (*Helianthus annuus* L.) and raw peanuts (*Arachishypogaea* L.) and a bouquet of branches of boldo (*Plectranthus barbatus* Andrews) placed in plastic pots with humidified vermiculite. The diet was replaced three times a week. Eggs were collected every two or three days and kept in plastic Petri dishes with a bean pod. When nymphs reached the second instar, they were transferred to 8 L containers and maintained following the same procedures as described for adults. Sexually mature virgin adults (≥10 days after the final molt) were used for the experiments [33,34]. Males and females were separated, by the external genitalia characters, after the imaginal molt and cuticular hardening (ca. 24 h after molting) and maintained in separated containers and rearing rooms until used in experiments.

### 2.2. Plants

All bioassays were conducted on bean plants (*Phaseolus vulgaris* L.). Beans were grown in plastic pots with a mixture of soil and organic growth substrate (1:1 *w*/*w*) and kept in a greenhouse (14 h L:10 h D). Experiments were conducted on bean plants with a 20 to 30 cm high stem and two fully expanded unifoliate leaves. 

### 2.3. Rival Signals and Stimulation Programs

For playback experiments, we used previously recorded rival signals from each species [10], selected from digital files (.wav recorded at 24-bit, 96- kHz, 100-dB signal-to-noise ratio, with a Sound Blaster Extigy, Creative Laboratories Inc., Milpitas, California, USA). The stimulation program consisted of sequences of rival signals produced by grouped females of each species (Figure S1). Signals from one to three different files were combined in a sequence with amplitude normalized to naturally emitted signals and at the same level within the whole sequence. The stimulation programs lasted 30 to 120 s, with 5 to 20 s of interval between signals from different individuals. For *C. impicticornis,* the last sequence of rivalry, when insects alternate FS-1b, which silences one of the rival females, was selected [10] (Figure S1). 

### 2.4. Playback Experimental Procedures

Experiments were conducted in a sound-proof room. Plants in pots were placed on a shock-proof table. Playback stimulation programs from each species were applied to bean plant surfaces by the tip of the 5 cm stick firmly fixed to the head of a vibration exciter (Mini-shaker Type 4810, Brüel & Kjaer, Naerum, Denmark), positioned horizontally on a polyurethane foam coated iron support. The mechanically isolated vibrator was in contact with the tested plant only by the tip of the stick, which was placed on the stalk ~10 cm above the soil level (Figure S2). Playback experiments were conducted in a random sequence of stimulation programs and insect species. A conspecific female and a male were placed individually on opposite leaves of the bean plant, which was vibrated (treatment) or not vibrated (control) with one of the stimulation programs of the respective species. Insects were observed for 20 min, monitoring their behavior and recording signals emitted during this period. 

Behavior categories recorded were the proportion of responses (number of pairs of each species emitting at least one signal in relation to total pairs tested), proportion of emissions of signals of each type by females and males in relation to the total females and males emitting signals, proportion of pair formation (number of pairs of each species in which the insects meet on the same leaf in relation to the total pairs tested), and proportion of copulation (number of pairs of each species that copulate in relation to total number of pairs tested). Latency (time from start the experiment until one of the insects of the pair starts to emit vibratory signals) and response time (time from start to emitting vibratory signals, until emitting the last one) were also registered.

Vibratory signals were recorded by a portable digital laser vibrometer (PDV-100, Polytec GmbH, Waldbronn, Germany). The laser beam was focused perpendicularly to a piece of a reflective tape of ~4 mm^2^ glued to the stalk of bean plants at ~20 cm from the soil surface at 2 to 3 cm below the insertion of unifoliate leaves, where the insects were placed. Surface vibrations, digitized by a sound card (24-bit, 96- kHz, 100-dB signal-to-noise ratio, Sound Blaster Extigy, Creative Laboratories Inc., Milpitas, CA, USA) were recorded and stored on a computer by Cool Edit Pro 2.0 software (Syntrillium Software 2001—Fort Wayne, Indiana, USA).

General experimental design included randomly reproduced stimulation programs of each species that were changed every three to five bioassays joint with the plants. For each species and condition of stimulation defined as treatment (plants vibrated with conspecific rival female signals) or control (non-vibrated plants), 25 to 30 repetitions were performed. Insects were considered as non-responsive if they did not emit any signals or displayed no reproductive behavior in the first 10 min of the bioassay. Insects that emitted signals were observed for 20 min or until they copulated. 

### 2.5. Signal Analyses

Female and male songs produced by abdomen vibration were classified and named according to Blassioli -Moraes et al. [8] for *E. heros* and Laumann et al. [12] for *C. ubica* and *C. impicticornis*. *E. heros* signals were identified as FS-1 (the first female song), FS-2 (the second female song), MS-1 (the first male song), MS-2 (the second male song). For *C. ubica* and *C. impicticornis,* signals were named FS-1a (the first female song, type a), FS-1b (the second female song, type b), MS-1 (the first male song) and MS-2 (the second male song). 

The basic units of vibratory emissions (pulses and pulse trains) [35] were described by their duration (ms) as the time between signal onset and end, repetition time (ms) as the time between onsets of two sequential pulses and/or pulse trains, and the number of pulses per pulse train. Sound Forge software (Sonic Foundry http://www.sonicfoundry.com) was used to analyze frequency spectra (Fast Fourier Transform (FFT) size 32,768, FFT overlap 99%, smoothing window Blackman–Harris, and display range 60–80 dB) and sonograms (FFT size 8192, FFT overlap 99%, smoothing window Blackman–Harris display range 40–80 dB). Spectra are described by the fundamental, dominant and harmonic peak frequencies. 

### 2.6. Statistical Analyses

Proportion of responses, proportion of emissions of signals of each type by females and males and proportion of copulation were compared between pairs of each species in treatment and control by logistic regression considering binary responses (yes—1 or no—0) as response variable, and control or treatment as explanatory factor. Coefficients and standard error from the logistic regressions were used to calculate the odds ratios (ORs) and their corresponding confidence interval of 95% (95% CI). Odds were considered significant if their CI did not include 1 value. The percentage of response reduction when insects were placed on vibrated plants was calculated as: 1-OR × 100, and the increase in response in non-vibrated plants (control) as: 1/OR. The proportion of pair formation, i.e., females and males on the same plant with visual and physical contact that were a consequence of males that showed oriented movements and reached female positions, was not directly observed and was estimated by computing the proportion between the number of insects that copulate in relation to the number of insects that emit vibratory signals. These proportions of males of each species with oriented movements on plants vibrated with rival songs (treatment) and on non-vibrated plants (control) were compared, using two-proportion z-tests with continuity corrections. 

Latency, response time and female and male vibratory signal temporal parameters (pulse duration, pulse train duration, repetition time of pulses or pulse train), number of pulses per pulse train and dominant frequency of normality distribution were evaluated with Shapiro–Wilk test. When data showed normal distribution, they were compared by repeated measure analyses of variance (ANOVA), considering the pulses measured in the same individual as repeated measures. When data did not show normality, they were analyzed with generalized linear mixed models (GLMMs) with Poisson distribution, considering the parameters as response variable, the condition in which insects were exposed (treatment or control) as explanatory factor and individuals as random effects. All statistical tests were developed in R platform version 4.0.0 (R Development Core Time, 2020) using the package lme4 for GLMM. Possible outliers identified in boxplot figures (Figure S2) were removed from the data set before the analyses. When models showed over-dispersion of data, a quasi GLM or quasi GLMM was used. Model diagnosis was evaluated by normal residues and quantile (qq-norm) plot curves (Figure S3). Scripts and results of statistical analyses are shown in the Supplementary File S1.

## 3. Results

### 3.1. Proportion of Responses

**Figure 1:**
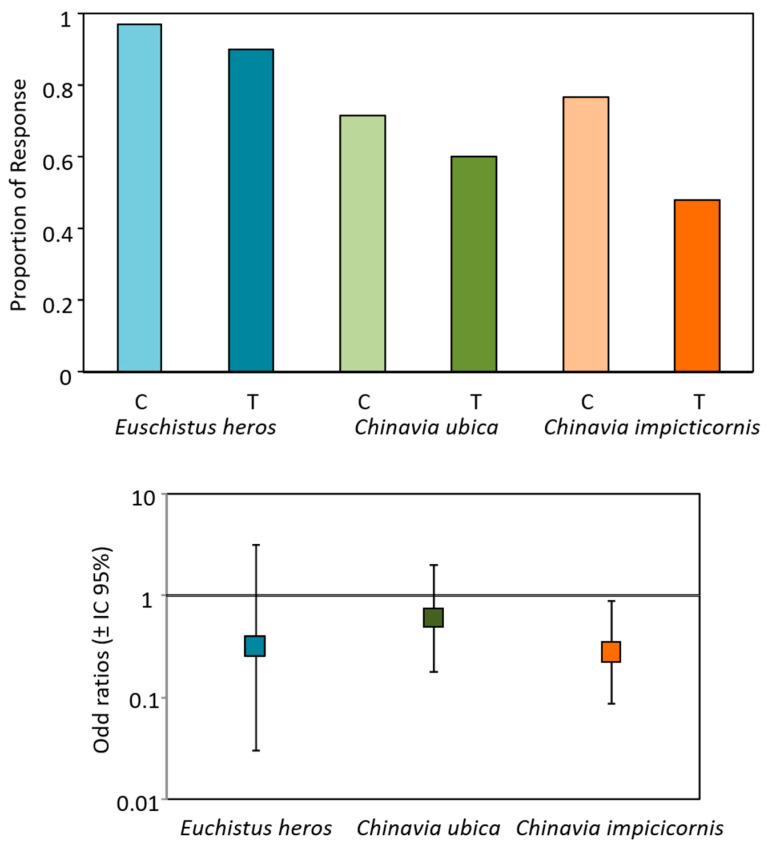
Effect of conspecific female rival signals on the emission of vibratory signals of three stink bug species. Upper graph: proportion of responses (number of pairs emitting vibratory signals/number of pairs tested) of *Euschistus heros*, *Chinaiva ubica* and *Chinaiva impicticornis* pairs (female and male) placed on control (non-vibrated—C) or treatment (vibrated with conspecific female rival signals—T). Lower graph: odds ratios (95% CI) = likelihood that a pair (female and male) on treatment plant will emit vibratory signals. Significance of odds ratios was established if 95% CI did not include 1. Proportions were calculated from 30 pairs of each species and treatment.

In the three species, a reduced proportion of pairs emitting vibratory signals was observed when submitted to playback of rival female signals, with significant effect only in *C. impicticornis* (*z* = −2.157, *p* = 0.031, df = 53, OR = 0.281, 95% CI = 0.089–0.891) (Figure 1 and Figure S1). In this case, the odds of pairs initiating vibratory communication increased by 3.55-fold in the absence of rival signals (control).

### 3.2. Latency and Response Time

**Figure 2:**
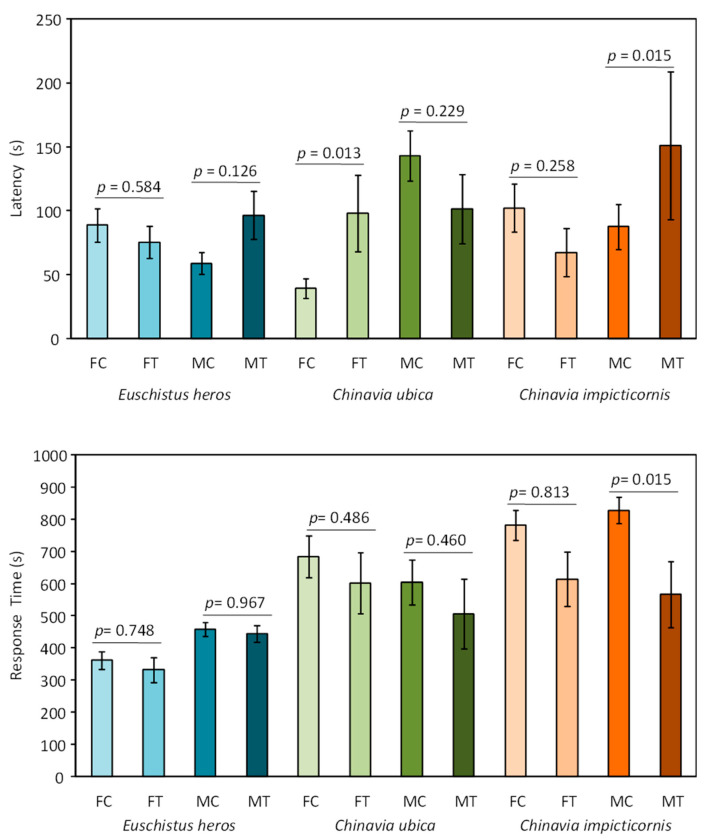
Effect of conspecific female rival signals on latency and response time of males and females of three stink bug species. Latency (s) (upper graph) and Response Time (s) (lower graph) of *Euschistus heros*, *Chinavia ubica* and *Chinavia impicticornis* females (F) and males (M) placed on control (non-vibrated—C) or treatment (vibrated with conspecific female rival signals—T) plants. Significant differences between times in C and T were established with generalized linear mixed models (GLLM). N = Latency *Euschistus heros* FC = 24, FT = 20, MC = 26, MT = 23, *Chinavia ubica* FC =14, FT = 10, MC = 17, MT = 11, *Chinavia impicticornis* FC = 23, FT = 11, MC = 22, MT = 9. Response time *Euschistus heros* FC = 29, FT = 24, MC = 27, MT = 21, *Chinavia ubica* FC =19, FT = 12, MC = 19, MT = 12, *Chinavia impicticornis* FC = 23, FT = 9, MC = 20, MT = 11.

Latency was affected only in *C. ubica* females, showing a longer time to start emission of vibratory signals when stimulated with conspecific female rival signals (*t* = 2.683, *p* = 0.013, df = 23) (Figure 2 and Figure S1). Only *C. impicticornis* males showed a shorter response time when stimulated by female rival signals (*t* = −2.592, *p* = 0.015, df = 29) (Figure 2).

### 3.3. Signal Emission and Parameters

**Figure 3:**
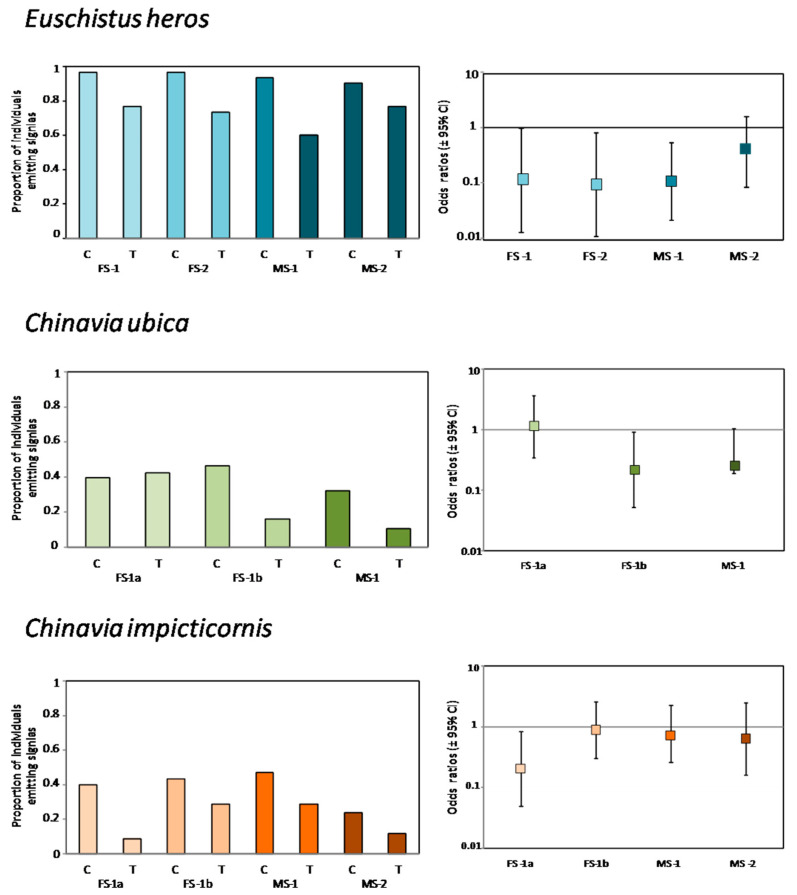
Effect of conspecific female rival signals on the emission of species specific vibratory signals of three stink bug species Left graphs: proportion of females (F) and males (M) of *Euschistus heros*, *Chinavia ubica* and *Chinavia impicticornis* emitting species specific signals when they were placed on control (non-vibrated—C) or treatment (vibrated with conspecific female rival signals—T) plants. Right graphs: odds ratios (95% CI) = likelihood of individuals (female or male) on treatment plant emitting species-specific vibratory signals. Significance of odds ratios was established if 95% CI did not include 1. Signals are named following Blassioli-Moraes et al. (2005) for *E. heros* and Laumann et al. (2016) for *C. ubica* and *C. impicticornis*. FS-1 = first female song, FS-2 = second female song 2, MS-1 = first male song 1. FS-1a = first female song type aa, FS-1b = first female song type b. N = *Euschsitus heros* C = 30, T = 30, *Chinavia ubica* C = 29, T = 20, *Chinavia impicticornis* C = 30, T = 25.

Proportion of *E. heros* individuals emitting the first (FS-1) and the second (FS-2) female song and the first (MS-1) male song was significantly reduced when insects were stimulated with rival female signals (treatment) (FS-1: *z* = −1.971, *p* = 0.048, df = 58, OR = 0.113, 95% CI = 0.012–0.988; FS-2: *z* = −2.146, *p* = 0.0032, df = 58, OR = 0.095, 95% CI = 0.011–0.815; MS-1: *z* = −2.719, *p* = 0.006, df = 58, OR = 0.107, 95% CI = 0.021–0.536) (Figure 3 and Figure S3). The odds of emitting FS-1, FS-2 and MS-1 increased 8.85, 10.52 and 9.34 times when insects were on non-vibrated (control) plants (Figure 3 and Figure S3). No significant differences were observed in the second male song (MS-2) when stimulated (vibrated plants) or in control (non-vibrated plants) conditions (Figure 3).

Table 1 b were significantly reduced in *Chinaviaubica* by female rival signals (*z* = −2.084, *p* = 0.037, df = 45, OR = 0.216, 95% CI = 0.051–0.913) (Figure 3). In this case, the odds of emitting FS-1b on non-vibrated plants were increased 4.63 times.

When *C. impicticornis* females and males were placed on plants vibrated with female rival signals, only FS-1a emissions were reduced in comparison with females placed on non-vibrated plants (*z* = −2.206, *p* = 0.027, df = 53, OR = 0.205, 95% CI = 0.049–0.838) (Figure 3). Odds of emitting FS-1b in non-vibrated plants were increased 4.88 times.

Playback of rival signals did not significantly affect the signal parameters emitted by *E. heros*. In this case, only dominant frequencies of female signals showed significant differences in relation to signals emitted by females in control plants (Table 1 and Table S1). In *C. ubica,* a significant effect was observed as an increasing pulse train repetition time of the first female song (FS-1a) and a reduction in the number of pulses per pulse train and an increase in dominant frequency of the second female song (FS-1b) when insects were on treated plants (Table 1 and Table S1). During stimulation, *C. impicticornis* females and males emitted signals with increasing temporal parameters (pulse train duration and pulse train repetition time) in FS-1a, a decrease in FS-1b pulse train repetition time and a decrease in pulse duration of the second male song (MS-2) in relation to signals emitted in control conditions (Table 1 and Table S1).

### 3.4. Proportion of Pair Formation and Copulation

**Figure 4:**
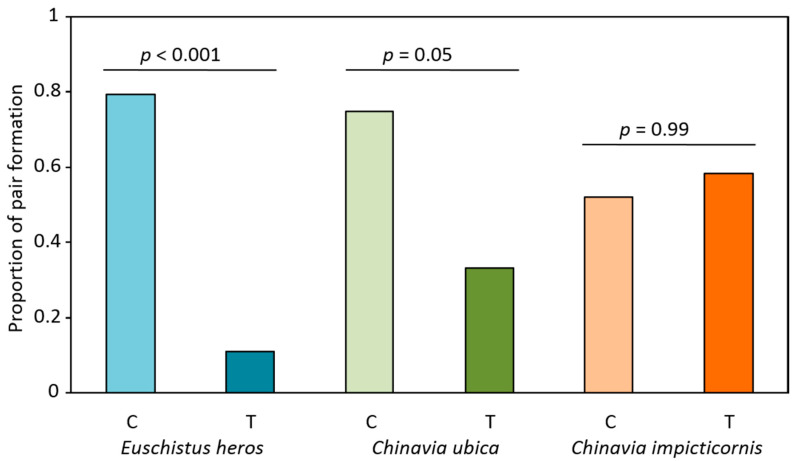
Effect of conspecific female rival signals on the pair formation (insects on the same the same leaf of the plant and in physical contact) of three stink bug species. Proportion of pair formation (individuals copulating/individuals emitting vibratory signals of *Euschistus heros*, *Chinavia ubica* and *Chinavia impicticornis* when they were placed on control (non-vibrated—C) or treatment (vibrated with conspecific rival female signals—T). Significant differences were established by z-test for two proportions with continuity corrections. N = *Euschistus heros* C = 29, T = 27, *Chinavia ubica* C = 20, T = 12, *Chinavia impicticornis* C = 23, T = 12.

The proportion of males that reached the female by oriented movement and formed a couple after exchanging vibratory signals was significantly lower in *E. heros* and *C. ubica* when stimulated by playback rival signals (χ^2^_1_ = 23.426, *p* < 0.001 and χ^2^_1_ = 3.809, *p* = 0.05). No significant differences were found in pair formation of *C. impicticornis* when they were placed on vibrated or non-vibrated plants (Figure 4).

**Figure 5:**
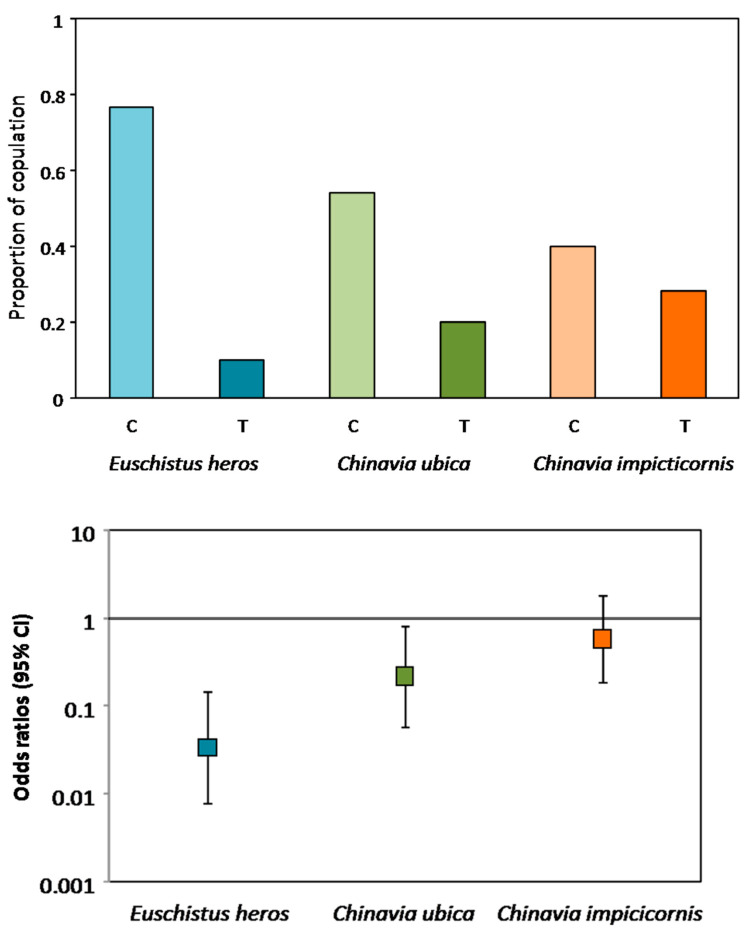
Effect of conspecific female rival signals on copulation of three stink bug species. Upper graph: proportion of pairs copulating (number of pairs copulating/number of pair tested) of *Euschistus heros*, *Chinavia ubica* and *Chinavia impicticornis* pairs (female and male) placed on control (non-vibrated—C) or treatment (vibrated with conspecific rival female signals—T). Lower graph: odds ratios (95% CI) = likelihood of a pair copulating on treatment plant. Significance of odds ratios was established if 95% CI did not include 1. N = *Euschistus heros* C = 23, T = 3, *Chinavia ubica* C = 15, T = 4, *Chinavia impicticornis* C = 13, T = 7.

A significant reduction in copulating pairs was observed when males and females of *E. heros* and *C. ubica* were placed on plants vibrated with conspecific rival female signals (*E. heros*: *z* = −4.539, *p* < 0.001, df = 58, OR = 0.034, 95% CI = 0.008–0.146, *C. ubica*: *z* = −2.265, *p* = 0.024, df = 46, OR = 0.217, 95% CI = 0.058–0.814) (Figure 5 and Figure S2). The likelihood of copulation in the absence of female rival signals increased 29.41 times in *E. heros*, 4.6 times in *C. ubica* and 1.71 times in *C. impicticornis*. However, in the last case, the difference in copulation proportions was not significant (*z* = −0.928, *p* = 0.313, df = 53) and odds ratios also did not show significance (OR = 0.583, 95% CI = 0.321–1.384) (Figure 5 and Figure S2).

## 4. Discussion

Results of the present study show a disrupting effect of rival female signals on the reproductive behavior of the three investigated stink bug species. This effect is more clearly expressed in *E. heros* and *C. ubica* than in *C. impicticornis*. In the first two species, we observed, from odds ratio analyses, a significant reduction in pair formation and copulation when insects were placed on plants vibrated with conspecific rival signals. This effect is associated with the reduction (expressed for odd ratios < 1) in specific vibratory communication signal emission (FS-1, FS-2 and MS-1 in *E. heros* and FS-1b in *C. ubica*), which may influence the duet formation and further development of different phases of mating behavior. Duetting by vibratory signals is decisive in stink bugs during the calling and courtship phase, working in pair formation and copulation [10]. In contrast with the latter two species, *C. impicticornis* rival female signals reduce the proportion of responsive insects (proportion of pairs that emit vibratory signals) but not pair formation or copulation. The different effects of rival signals in the tested species could be related to their difference in rivalry behavior, which is associated with differences in the vibratory signal structure. *E. heros* and *C. ubica* emit specific rival signals (FRS) after alternation with female calling signals (FS-1 of *E. heros* and FS-1a and FS-1b for *C. ubica*). In contrast, rivalry in *C. impicticornis* is expressed by alternation and transition of calling song FS-1a to the FS-1b type, which is maintained until one of the competitors falls silent [10]. Considering these specific characteristics, we may hypothesize that rival signals of *E. heros* and *C. ubica* inhibit conspecifics more than those of *C. impicticornis*. This is also supported by the observation that the proportions of insect signaling, latency and response time of *C.ubica* and *E. heros* were not significantly affected by rival female signals played back to plants, but in both cases the number of pairs copulating was reduced in the presence of this signals.

In *C. impicticornis* the proportion of pairs that copulate was not reduced by female rival signals and may be a consequence of some mechanisms of adaptation to a noisy background by modifying the spectral and temporal parameters of their signals. In general, *C. impicticornis* individuals extend the duration of calling signals (FS-1a) and shorten pulses and repetition time of duet signals (FS-1b and MS-1) when in the presence of rival female signals. A similar variation in signal emissions was observed in females of *N. viridula* during female rivalry interactions [11] that, similarly to *C. impicticornis*, proceed in different levels of complexity by the emission of three types of rival songs [11]. However, as temporal parameters of stink bug signals are directly related to gender and species-specificity, and characteristics need to be conserved during communication [8,12,36,37,38], it was observed that *C. impicticornis* signal parameters of individuals in noisy environments are in the general species-specific range [12].

Several studies have showed inhibitory and disrupting effects on airborne or substrate-borne communication by environmental noise [39,40,41], and the application of this knowledge to behavioral pest management has been proposed [14]. One of the proposed strategies is using vibrations as a tool to disrupt mating [18,19]. This strategy was successfully tested in different hemipterans, such as Cicadellidae, Delphacidae, Liviidae and Triozidae, using natural (e.g., disruptive, rival or female signals) or artificially synthesized (ex. white noise, pure tone) vibrations [17,18,19,23,24,42,43,44]. The general pattern observed in these studies suggests that mating disruption could be achieved by interference in communication, which reduces signal emission and the probability of pair formation. A similar effect was observed in our study on *E. heros* and *C. ubica*.

Mating disruption in stink bugs was studied first in *E. heros* using continuous pure tone vibrations as interference background noise [25]. Playing back pure-tone vibrations (75 to 200 Hz) showed significant effects on *E. heros* communication, reducing the responses of males to calling signals and their search for females. In addition, this pure-tone vibration had a strong effect on copulation, reducing it by 94.2 to 100% in relation to control pairs [25]. However, in long-duration experiments (24 h), the reduction in copulation was lower and reached 24.7% on plants with background noise in relation to results obtained in experiments on non-stimulated plants [25]. Similar results obtained in experiments with playback rival female signals showed a reduction in copulation as a result of reducing signals and duetting emissions and of disrupting male directional movement to calling females. 

The disrupting effect of background noise on males searching for females was also shown in *N. viridula* [45]. In this species, searching and orientation were also negatively affected by simultaneous playback of female calling songs from conspecific and alien species [37]. The principal mechanism for vibrational directionality is detection and processing of amplitude, phase and/or time differences in the time of signals arrival at leg receptors spatially distributed on the substrate [46,47]. Background vibrations could disrupt stink bugs directionality by interference with naturally emitted signals, breaking the differences in amplitude, phase or time.

The reduction in the efficiency of background noise (emitted as pure-tone vibrations) in disrupting mating in long-duration experiments in *E. heros* was explained as result of habituation [25]. It was proposed that this habituation effect could be reduced with different strategies, for example, the use of discontinuous reproduction of artificial signals or of predators, rival or male signals emitted prior to copulation [25], because all of these signals have disruptive effects on stink bug communication [10]. Following this hypothesis, we tested the impact of rival signals. The results presented here suggest their potential for application in pest control management as a tool for manipulation and disruption of mating behavior. Further work needs to focus on the effects of rival female signals used in long-duration experiments and on their interference in the release of the male sex pheromone. Stink bugs’ sexual behavior includes communication with signals of different modalities, principally chemicals (pheromone) and substrate-borne vibration [48]. The mating disruption strategy needs to consider interactions (e.g., synergistic effects and regulation of signal emission by signals from another modality) between signals of these two modalities.

The use of a mating disruption strategy at the present stage of technology seems to be more complicated in crops in large areas. This is the case of soybean in Brazil and other countries [15], compared with previously studied systems such as those described for vineyard pests, that use an electromagnetic vibrator coupled to the wires used to support the plants [18,20,22,43] and for *D. citri*, where disturbing synthetic female responses are played back with a microcontroller piezo buzzer platform after detecting a male calling [23,24]. However, the combined use of sex pheromones and disrupting signals could be used in a mating disruption strategy in extensive crop areas. Pheromones could be used to aggregate insects in specific places in the cultivated fields, where the disrupting signals could be applied successfully by mechanical or airborne components that transmit them to plants. 

## 5. Conclusions

Vibratory communication among stink bugs offers a great opportunity to develop biorational pest control tools based on their behavioral manipulation, and mating disruption could be one of the more promising strategies for this. In this work, it was demonstrated at first that (1) female rival signals played back as background noise affect vibratory communication of pairs of three stink bug species. In two cases (*E. heros* and *C. ubica*), female rival signals silence the pair, interrupting the duet signalization. In the other species, *C. impicticornis*, insects in the presence of female rival signals change temporal and spectral parameters to avoid background noise interference (Hypothesis 1); (2) as a consequence of this, the presence of rival signals reduces the proportion of pairs developing mating behavior and copulation. This effect was more strongly observed in *E. heros* and *C. ubica* (Hypothesis 2).

The results show potential for interference in stink bugs communication and for disrupting mating. The effect on population dynamics and pest control needs to be tested in long duration semi-field and field experiments.

## Figures and Tables

**Table 1:** Temporal and spectral characteristics of *Euschitus heros*, *Chinavia ubica* and *C. impicticornis* females and males when recorded from couples on bean plants without vibrations or vibrated with rival female signals from conspecifics in playback experiments.

Species	Control	Treatment	Statistics
***Euschistus heros***			
**FS-1**			
PD	1096.62 ± 325.10	985.34 ± 320.40	z = −1.63, *p* = 0.104
RT	2332.98 ± 1472.11	2034.28 ± 1396.36	z = −1.64, *p* = 0.219
DF	115.08 ± 9.36	120.13 ± 11.71	**z = 2.13, *p* = 0.033**
N/Ni	314/30	229/23	
**FS-2**			
PD	3506.61 ± 1372.20	3437.11 ± 1420.73	z = −1.43, *p* = 0.152
DF	118.75 ± 8.35	125.58 ± 10.58	**z = 2.60, *p* = 0.009**
N/Ni	169/29	62/22	
**MS-1**			
PD	1613.01 ± 657.37	1667.63 ± 737.22	z = 0.82, *p* = 0.410
RT	3394.05 ± 2279.39	2560.49 ± 1837.38	z = −1.74, *p* = 0.082
DF	142.62 ± 13.98	140.86 ± 15.68	z = 0.04, *p* = 0.970
N/Ni	212/28	78/18	
**MS-2**			
PD	5156.69 ± 2077.66	4856.28 ± 1693.33	z = −1.19, *p* = 0.232
DF	138.59 ± 11.33	144.82 ± 15.44	z = 0.79, *p* = 0.432
N/Ni	141/27	173/23	
***Chinavia ubica***			
**FS-1a**			
PTD	1707.71 ±440.34	1923.32 ± 505.19	z = 1.28, *p* = 0.209
NPPT	9.32 ± 2.23	10.03 ± 2.19	z = 0.97, *p* = 0.333
PTRT	4051.17 ± 1294.39	4700.43 ± 1958.94	**z = 1.78, *p* = 0.075**
DF	103.10 ± 7.55	108.25 ± 5.72	
N/Ni	41/10	53/8	
**FS-1b**			
PTD	1063.16 ± 205.65	879.95 ± 117.72	t = −2.09, *p* = 0.097
NPPT	4.95 ± 1.45	3.35 ± 1.35	**z = −2.74, *p* = 0.006**
PTRT	2251.95 ± 275.26	2271.90 ± 1329.45	z = 0.24, *p* = 0.807
DF	100.45 ± 7.61	107.60 ± 2.85	**z = 2.55, *p* = 0.011**
N/Ni	38/8	20/3	
**MS-1**			
PTD	1808.84 ± 440.94	2144.02 ± 655.45	z = 1.36, *p* = 0.186
NPPT	9.44 ± 2.41	7.19 ± 1.36	z = −0.95, *p* = 0.343
DF	106.12 ± 5.33	113.81 ± 6.12	z = 0.39, *p* = 0.692
N/Ni	50/9	21/2	
***Chinavia impicticornis***			
**FS-1a**			
PTD	3188.03 ± 549.07	4442.93 ± 334.44	**z = 3.20, *p* = 0.001**
NPPT	12.77 ± 3.47	16.36 ± 1.01	**z = 2.37, *p* = 0.018**
PTRT	7358.78 ± 3395.57	9675.53 ± 1016.06	z = 1.72, *p* = 0.086
DF	87.30 ± 9.55	93.43 ± 3.63	z = 1.44, *p* = 0.149
N/Ni	105/10	15/3	
**FS-1b**			
PTD	1193.23 ± 298.66	1040.14 ± 230.27	z = −1.32, *p* = 0.186
NPPT	2.30 ± 0.96	2.32 ± 0.99	z = −0.08, *p* = 0.933
PTRT	4307.58 ± 1794.54	3007.68 ± 740.51	**z = −3.64, *p* = 0.0002**
DF	88.39 ± 4.74	90.65 ± 5.46	z = 1.08, *p* = 0.280
N/Ni	119/11	168/11	
***C. impicticornis***			
**MS-1**			
PTD	3957.88 ± 622.39	3531.72 ± 755.82	**t = −2.41, *p* = 0.024**
NPPT	16.34 ± 2.57	14.92 ± 2.94	**z = −2.31, *p* = 0.020**
DF	87.93 ± 7.81	96.12 ± 6.40	z = 1.28, *p* = 0.209
N/Ni	134/14	158/15	
**MS-2**			
PD	185.83 ± 35.94	148.36 ± 33.74	**z = −2.26, *p* = 0.024**
PRT	265.72 ± 44.35	230.27 ± 26.38	z = −1.83, *p* = 0.068
DF	99.93 ± 13.33	107.25 ± 7.07	z = 1.03, *p* = 0.293

References: Signals are named following Blassioli-Moraes et al. (2005) [8] for *Euschistus heros* and Laumann et al. (2016) [12] for *Chinavia ubica* and *Chinavia impicticornis*. FS-1 = first female song, FS-2 = second female song 2, MS-1 = first male song 1. FS-1a = first female song type aa, FS-1b = first female song type b. PD = pulse duration (ms), RT: repetition time (ms), DF = dominant frequency (Hz), PTD = pulse train duration (ms), NPPT = number of pulses/pulse train, PTRT = pulse train repetition time, N = number of individual signals, Ni = number of individuals.

## Data Availability

The data has not been presented in a separate link but can be made available to anyone interested by contacting the corresponding author.

## Supplementary Materials

The following are available online at https://www.mdpi.com/2075-4450/12/2/177/s1, Figure S1: Oscillograms of stimulation programs used in the play back experiments. Figures show a complete sequence of pulses (with the total duration at right) of one stimulation programs for each species studied and a detail (marked in the stimulation program with a red square) of a sequence of pulses with the correspondent one second scale. Euschistus heros: sequences of rival songs of three different female rival interactions. Chinavia ubica: a long sequence of rival songs from one female rival interaction. Chinavia impicticornis: sequence of alternation of FS-1b of two different female rival interactions. Figure S2: General setup of the play back experiments. File S1: Statistical analyses scripts and results.
