# Supplementary material for: Inhibitory Copulation Effect of Vibrational Rival Female Signals of Three Stink Bug Species as a Tool for Mating Disruption
Source: Insects. 2021 Feb 18;12(2):177. doi: 10.3390/insects12020177 (PMC7923018; doi:10.3390/insects12020177)
Supplement: Supplementary file 1 [file insects-12-00177-s001.zip › insects-1062341 Supplementary Material/inects-1062341 Figure S1.pdf]

Figure S1

Inhibitory copulation effect of vibrational rival female signals of three stink bug species as a tool for mating disruption

Aline Moreira Dias, Miguel Borges, Maria Carolina Blassioli Moraes, Matheus Lorran Figueira Coelho, Andrej Čokl, Raul Alberto Laumann

Figure S1. Oscillograms of stimulation programs used in the play back experiments. Figures show a complete sequence of pulses (with the total duration at right) of one stimulation programs for each species studied and a detail (marked in the stimulation program with a red square) of a sequence of pulses with the correspondent one second scale. *Euschistus heros*: sequences of rival songs of three different female rival interactions. *Chinavia ubica*: a long sequence of rival songs from one female rival interaction. *Chinavia impicticornis*: sequence of alternation of FS-1b of two different female rival interactions.

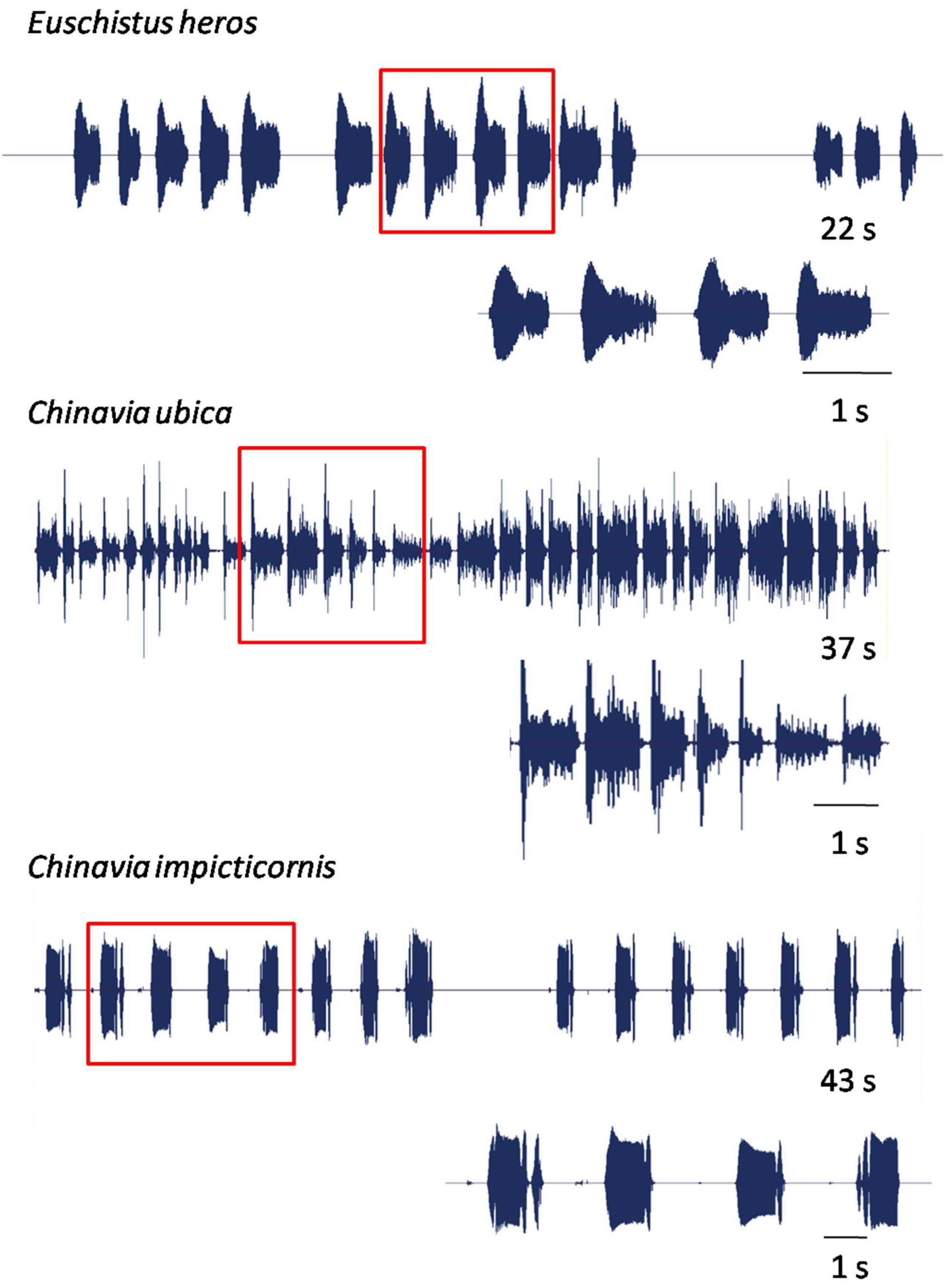

Euschistus heros
22 s
1 s
Chinavia ubica
37 s
1 s
Chinavia impicticornis
43 s
1 s
