# Supplementary material for: Inhibitory Copulation Effect of Vibrational Rival Female Signals of Three Stink Bug Species as a Tool for Mating Disruption
Source: Insects. 2021 Feb 18;12(2):177. doi: 10.3390/insects12020177 (PMC7923018; doi:10.3390/insects12020177)
Supplement: Supplementary file 1 [file insects-12-00177-s001.zip › insects-1062341 Supplementary Material/Dias et al 2020 S1.pdf]

Supplementary material S1

Inhibitory copulation effect of vibrational rival female signals of three stink bug species as a tool for mating disruption

Aline Moreira Dias, Miguel Borges, Maria Carolina Blassioli Moraes, Matheus Lorran Figueira Coelho, Andrej Čokl, Raul Alberto Laumann

Statistical analyses scripts and results

Euschistus heros

Proportion of Responses

```
> analise=glm(Ehresp~Eh, family=binomial)
> summary(analise)

Call:
glm(formula = Ehresp ~ Eh, family = binomial)

Deviance Residuals:
    Min       1Q   Median       3Q      Max
-2.6081   0.2604   0.2604   0.4590   0.4590

Coefficients:
            Estimate Std. Error z value Pr(>|z|)
(Intercept)    3.367      1.017   3.311 0.000931 ***
EhEht         -1.170      1.185  -0.987 0.323554
(Dispersion parameter for binomial family taken to be 1)

    Null deviance: 29.392  on 59  degrees of freedom
Residual deviance: 28.274  on 58  degrees of freedom
AIC: 32.274
Number of Fisher Scoring iterations: 6

> anova(analise, test="Chi")
Analysis of Deviance Table

Model: binomial, link: logit
Response: Ehresp
Terms added sequentially (first to last)

     Df Deviance Resid. Df Resid. Dev Pr(>Chi)
NULL                    59     29.392
Eh    1   1.1179        58     28.274   0.2904

> odd.ratio = exp(coef(analise));odd.ratio
(Intercept)      EhEht
 29.0000000  0.3103448
> ICbeta1=confint.default(analise,level=0.95);ICbeta1
                 2.5 %    97.5 %
(Intercept)  1.373827   5.360765
EhEht       -3.493147   1.153004
> ICOR1=exp(ICbeta1);ICOR1
                 2.5 %     97.5 %
(Intercept) 3.95044019 212.887668
EhEht       0.03040504   3.167696
```

Female

Latency

```
> dados=read.table("tlehf.txt", h=T)

> shapiro.test(TL)

        Shapiro-Wilk normality test
data:  TL
W = 0.94521, p-value = 0.0366
```

```
> analise=glm(TL~Femea, family=quasipoisson)
> summary(analise)
Call:
glm(formula = TL ~ Femea, family = quasipoisson)
Deviance Residuals:
    Min       1Q    Median       3Q      Max
-11.8817   -4.6255   -0.9545    3.4977   12.2313

Coefficients:
                     Estimate Std. Error t value Pr(>|t|)
(Intercept)          4.3296     0.1355  31.960   <2e-16 ***
FemeaTreatment  -0.1146     0.2075  -0.552    0.584
(Dispersion parameter for quasipoisson family taken to be 33.43867)
    Null deviance: 1473.9  on 43  degrees of freedom
Residual deviance: 1463.6  on 42  degrees of freedom
AIC: NA
Number of Fisher Scoring iterations: 5
```

Response time

```
> dados=read.table("tcehf.txt", h=T)
> shapiro.test(RT)

        Shapiro-Wilk normality test
data:  RT
W = 0.93607, p-value = 0.007062

> analise=glm(RT~Femea, family=quasipoisson)
> summary(analise)
Call:
glm(formula = RT ~ Femea, family = quasipoisson)

Deviance Residuals:
    Min      1Q   Median      3Q     Max
-19.536   -5.453    2.033    6.390   11.114

Coefficients:
                     Estimate Std. Error t value Pr(>|t|)
(Intercept)          5.91917    0.07957  74.391   <2e-16 ***
FemeaTreatment -0.03864    0.11951  -0.323    0.748
(Dispersion parameter for quasipoisson family taken to be 68.31945)
    Null deviance: 4063.2  on 52  degrees of freedom
Residual deviance: 4056.1  on 51  degrees of freedom
AIC: NA
Number of Fisher Scoring iterations: 4
```

Male

Latency

```
> dados=read.table("tlehm.txt", h=T)
> shapiro.test(TL)

        Shapiro-Wilk normality test
data:  TL
W = 0.86322, p-value = 4.228e-05

> analise=glm(TL~Macho, family=quasipoisson)
> summary(analise)

Call:
glm(formula = TL ~ Macho, family = quasipoisson)
```

```
Deviance Residuals:
     Min        1Q    Median        3Q       Max
-11.5671   -7.0145   -0.7657    3.1020   14.8913

Coefficients:
                  Estimate Std. Error t value Pr(>|t|)
(Intercept)         3.9961     0.1848  21.627   <2e-16 ***
MachoTreatment   0.3827     0.2459   1.556    0.126

(Dispersion parameter for quasipoisson family taken to be 48.27374)
    Null deviance: 2415.7  on 48  degrees of freedom
Residual deviance: 2297.5  on 47  degrees of freedom
AIC: NA
```

Response time

```
> shapiro.test(TC)
        Shapiro-Wilk normality test
data:  TC
W = 0.97067, p-value = 0.2689

> t.test(TC~Macho)

        Welch Two Sample t-test
data:  TC by Macho
t = -0.041954, df = 45.185, p-value = 0.9667
alternative hypothesis: true difference in means is not equal to 0

95 percent confidence interval:
 -57.55812  55.20892
sample estimates:
  mean in group Control mean in group Treatment
               470.1111                471.2857
```

Proportion of couple formation

```
> res = prop.test(x = c(23, 3), n = c(29, 27))
> res

        2-sample test for equality of proportions with continuity correction

data:  c(23, 3) out of c(29, 27)
X-squared = 23.476, df = 1, p-value = 1.265e-06
alternative hypothesis: two.sided

95 percent confidence interval:
 0.4570551 0.9069296


sample estimates:
   prop 1    prop 2
0.7931034 0.1111111
```

Proportion of copulation

```
> analise=glm(Ehcop~Eh, family=binomial)
> summary(analise)

Call:
glm(formula = Ehcop ~ Eh, family = binomial)
```

```
Deviance Residuals:
   Min      1Q  Median      3Q     Max
-1.706  -0.459  -0.459   0.729   2.146

Coefficients:
            Estimate Std. Error z value Pr(>|z|)
(Intercept)   1.1896     0.4317   2.756  0.00585 **
EhEht        -3.3868     0.7461  -4.539 5.64e-06 ***
(Dispersion parameter for binomial family taken to be 1)
    Null deviance: 82.108  on 59  degrees of freedom
Residual deviance: 52.101  on 58  degrees of freedom
AIC: 56.101

Number of Fisher Scoring iterations: 4

> anova(analise, test="Chi")
Analysis of Deviance Table
Model: binomial, link: logit
Response: Ehcop
Terms added sequentially (first to last)

     Df Deviance Resid. Df  Resid. Dev  Pr(>Chi)
NULL                    59     82.108
Eh    1   30.006        58     52.101    4.306e-08 ***

> odd.ratio = exp(coef(analise));odd.ratio
(Intercept)       EhEht
 3.28571429  0.03381643
> ICbeta1=confint.default(analise,level=0.95);ICbeta1
                 2.5 %      97.5 %
(Intercept)  0.3435352    2.035633
EhEht       -4.8491105   -1.924507
> ICOR1=exp(ICbeta1);ICOR1
                  2.5 %      97.5 %
(Intercept) 1.409923154  7.6570970
EhEht       0.007835344  0.1459477
```

Signals

FS-1

```
> dados=read.table("eh.txt", h=T)
> attach(dados)
> names(dados)
[1] "trat" "FS1"  "FS2"  "MS1"  "MS2"
> analise=glm(FS1~trat, family=binomial)
> summary(analise)

Call:
glm(formula = FS1 ~ trat, family = binomial)

Deviance Residuals:
    Min       1Q   Median       3Q      Max
-2.6081   0.2604   0.2604   0.7290   0.7290

Coefficients:
            Estimate Std. Error   z value    Pr(>|z|)
(Intercept)    3.367      1.017     3.311    0.000931 ***
tratT         -2.178      1.105    -1.971    0.048730 *
(Dispersion parameter for binomial family taken to be 1)

    Null deviance: 47.121  on 59  degrees of freedom
```

Residual deviance: 41.365 on 58 degrees of freedom
AIC: 45.365

Number of Fisher Scoring iterations: 6

> anova(analise, test="Chi")
Analysis of Deviance Table

Model: binomial, link: logit
Response: FS1
Terms added sequentially (first to last)

	Df	Deviance	Resid. Df	Resid. Dev	Pr(>Chi)
NULL			59	47.121	
trat	1	5.7559	58	41.365	0.01643 *

> odd.ratio = exp(coef(analise));odd.ratio

(Intercept)	tratT
29.0000000	0.1133005

> ICbeta1=confint.default(analise,level=0.95);ICbeta1

	2.5 %	97.5 %
(Intercept)	1.373827	5.36076465
tratT	-4.343287	-0.01213616

> ICOR1=exp(ICbeta1);ICOR1

	2.5 %	97.5 %
(Intercept)	3.95044019	212.8876683
tratT	0.01299374	0.9879372

FS-2

> analise=glm(FS2~trat, family=binomial)
> summary(analise)

Call:
glm(formula = FS2 ~ trat, family = binomial)
Deviance Residuals:

Min	1Q	Median	3Q	Max
-2.6081	0.2604	0.2604	0.7876	0.7876

Coefficients:

	Estimate	Std. Error	z value	Pr(>|z|)
(Intercept)	3.367	1.017	3.311	0.000931 ***
tratT	-2.356	1.098	-2.146	0.031870 *

(Dispersion parameter for binomial family taken to be 1)
Null deviance: 50.725 on 59 degrees of freedom
Residual deviance: 43.564 on 58 degrees of freedom
AIC: 47.564
Number of Fisher Scoring iterations: 6
> anova(analise, test="Chi")
Analysis of Deviance Table

Model: binomial, link: logit

Response: FS2

Terms added sequentially (first to last)

	Df	Deviance	Resid. Df	Resid. Dev	Pr(>Chi)
NULL			59	50.725	

```
trat            1   7.1615                   58     43.564    0.007449 **

> odd.ratio = exp(coef(analise));odd.ratio
(Intercept)       tratT
29.00000000  0.09482759
> ICbeta1=confint.default(analise,level=0.95);ICbeta1
              2.5 %     97.5 %
(Intercept)  1.373827  5.3607646
tratT       -4.507139 -0.2042507
> ICOR1=exp(ICbeta1);ICOR1
                    2.5 %           97.5 %
(Intercept) 3.95044019   212.8876683
tratT         0.01102997   0.8152579
```

MS-1

```
> analise=glm(MS1~trat, family=binomial)
> summary(analise)

Call:
glm(formula = MS1 ~ trat, family = binomial)

Deviance Residuals:
    Min       1Q   Median       3Q      Max
-2.3272   0.3715   0.3715   1.0108   1.0108

Coefficients:
                Estimate Std. Error     z value      Pr(>|z|)
(Intercept)   2.6391      0.7319        3.606        0.000311 ***
tratT          -2.2336      0.8213        -2.719       0.006539 **

(Dispersion parameter for binomial family taken to be 1)

    Null deviance: 65.193  on 59  degrees of freedom
Residual deviance: 55.077  on 58  degrees of freedom
AIC: 59.077

Number of Fisher Scoring iterations: 5

> anova(analise, test="Chi")
Analysis of Deviance Table

Model: binomial, link: logit
Response: MS1
Terms added sequentially (first to last)

                Df Deviance Resid.   Df   Resid. Dev Pr(>Chi)
NULL                                 59     65.193
trat            1     10.116         58     55.077  0.00147 **

> odd.ratio = exp(coef(analise));odd.ratio
(Intercept)       tratT
 14.0000000   0.1071429
> ICbeta1=confint.default(analise,level=0.95);ICbeta1
              2.5 %    97.5 %
(Intercept)  1.204515  4.073599
tratT       -3.843389 -0.623795
> ICOR1=exp(ICbeta1);ICOR1
                    2.5 %          97.5 %
(Intercept) 3.33514195  58.7681133
tratT         0.02142087     0.5359068
```

MS-2

```
> analise=glm(MS2~trat, family=binomial)
> summary(analise)

Call:
glm(formula = MS2 ~ trat, family = binomial)

Deviance Residuals:
   Min      1Q  Median     3Q     Max
-2.146   0.459   0.459   0.729   0.729

Coefficients:
             Estimate  Std. Error  z value    Pr(>|z|)
(Intercept)   2.1972    0.6085      3.611     0.000305 ***
tratT        -1.0076    0.7461     -1.351     0.176834

(Dispersion parameter for binomial family taken to be 1)
    Null deviance: 54.067  on 59  degrees of freedom
Residual deviance: 52.101  on 58  degrees of freedom
AIC: 56.101

Number of Fisher Scoring iterations: 4

> anova(analise, test="Chi")
Analysis of Deviance Table

Model: binomial, link: logit
Response: MS2
Terms added sequentially (first to last)


          Df Deviance Resid.  Df    Resid. Dev Pr(>Chi)
NULL                          59     54.067
trat       1    1.966         58     52.101      0.1609
> odd.ratio = exp(coef(analise));odd.ratio
(Intercept)      tratT
  8.9999998   0.3650794
> ICbeta1=confint.default(analise,level=0.95);ICbeta1
              2.5 %    97.5 %
(Intercept)  1.004527 3.3899224
tratT       -2.469942 0.4546614
> ICOR1=exp(ICbeta1);ICOR1
                 2.5 %      97.5 %
(Intercept) 2.73061474   29.66365
tratT       0.08458974   1.57564
```

Chinavia ubica

Proportion of response

```
> analise=glm(Curesp~Cu, family=binomial)
> summary(analise)
Call:
glm(formula = Curesp ~ Cu, family = binomial)
Deviance Residuals:
    Min      1Q   Median      3Q      Max
-1.5829  -1.3537   0.8203   0.8679   1.0108
```

```
Coefficients:
            Estimate Std. Error  z value Pr(>|z|)
(Intercept)   0.9163     0.4183    2.190      0.0285 *
CuCut        -0.5108     0.6191   -0.825      0.4093
(Dispersion parameter for binomial family taken to be 1)
    Null deviance: 61.105  on 47  degrees of freedom
Residual deviance: 60.424  on 46  degrees of freedom
AIC: 64.424

Number of Fisher Scoring iterations: 4

> anova(analise, test="Chi")
Analysis of Deviance Table
Model: binomial, link: logit
Response: Curesp
Terms added sequentially (first to last)

          Df Deviance Resid. Df   Resid. Dev   Pr(>Chi)
NULL                         47     61.105
Cu         1     0.6818       46    60.424        0.409
> odd.ratio = exp(coef(analise));odd.ratio
(Intercept)      CuCut
       2.5        0.6
> ICbeta1=confint.default(analise,level=0.95);ICbeta1
                 2.5 %    97.5 %
(Intercept)  0.0963795 1.7362020
CuCut       -1.7243158 0.7026645
> ICOR1=exp(ICbeta1);ICOR1
                  2.5 %      97.5 %
(Intercept) 1.101177    5.675746
CuCut       0.178295    2.019126
```

Female

Latency

```
> dados=read.table("tlcuf.txt", h=T)
> shapiro.test(TL)

        Shapiro-Wilk normality test
data:  TL
W = 0.78299, p-value = 0.0001198

> analise=glm(TL~Femea, family=quasipoisson)
> summary(analise)

Call:
glm(formula = TL ~ Femea, family = quasipoisson)

Deviance Residuals:
    Min       1Q   Median       3Q      Max
-10.229   -3.010   -1.110    1.929   10.182

Coefficients:
                  Estimate Std. Error t value Pr(>|t|)
(Intercept)         3.3964     0.2584  13.146  3.5e-12 ***
FemeaTreatment      0.8598     0.3205   2.683   0.0133 *

(Dispersion parameter for quasipoisson family taken to be 27.89983)
    Null deviance: 867.81  on 24  degrees of freedom
Residual deviance: 655.15  on 23  degrees of freedom
AIC: NA
```

Number of Fisher Scoring iterations: 5

Response time

```
> dados=read.table("tccuf.txt", h=T)
> shapiro.test(RT)

        Shapiro-Wilk normality test
data:  RT
W = 0.95747, p-value = 0.2497

> t.test(RT~Femea)

        Welch Two Sample t-test

data:  RT by Femea
t = 0.70938, df = 20.841, p-value = 0.486

alternative hypothesis: true difference in means is not equal to 0
95 percent confidence interval:
 -157.9939  321.4676
sample estimates:
  mean in group Control mean in group Treatment
             683.7368                602.0000
```

Males

Latency

```
> dados=read.table("tlcum.txt", h=T)
> attach(data)
> names(dados)
[1] "Machos" "TL"
> shapiro.test(TL)

        Shapiro-Wilk normality test

data:  TL
W = 0.9445, p-value = 0.1439

> t.test(TL~Machos)

        Welch Two Sample t-test

data:  TL by Machos
t = 1.2426, df = 19.744, p-value = 0.2286

alternative hypothesis: true difference in means is not equal to 0
95 percent confidence interval:
 -28.27341 111.41780
sample estimates:
  mean in group Control mean in group Treatment
             143.1176                101.5455
```

Response time

```
> dados=read.table("tccum.txt", h=T)
> shapiro.test(RT)

        Shapiro-Wilk normality test
data:  RT
W = 0.93658, p-value = 0.06638
```

```
> t.test(RT~Machos)

        Welch Two Sample t-test

data:  RT by Machos
t = 0.7539, df = 20.009, p-value = 0.4597

alternative hypothesis: true difference in means is not equal to 0
95 percent confidence interval:
 -171.8785  366.4400
sample estimates:
  mean in group Control mean in group Treatment
               603.9474                506.6667
```

Proportion of couple formation

```
> res = prop.test(x = c(15, 4), n = c(20, 12))
> res
        2-sample test for equality of proportions with continuity correction

data:  c(15, 4) out of c(20, 12)
X-squared = 3.8089, df = 1, p-value = 0.05098
alternative hypothesis: two.sided
95 percent confidence interval:
 0.0226595 0.8106738
sample estimates:
   prop 1    prop 2
0.7500000 0.3333333
```

Proportion of copulation

```
> analise=glm(Cucop~Cu, family=binomial)
> summary(analise)

Call:
glm(formula = Cucop ~ Cu, family = binomial)
Deviance Residuals:
    Min       1Q   Median       3Q      Max
-1.2388  -1.2388  -0.6681   1.1173   1.7941

Coefficients:
            Estimate Std. Error  z value  Pr(>|z|)
(Intercept)   0.1431     0.3789    0.378    0.7057
CuCut        -1.5294     0.6753   -2.265    0.0235 *
(Dispersion parameter for binomial family taken to be 1)
    Null deviance: 64.443  on 47  degrees of freedom
Residual deviance: 58.689  on 46  degrees of freedom
AIC: 62.689
Number of Fisher Scoring iterations: 4

> anova(analise, test="Chi")
Analysis of Deviance Table
Model: binomial, link: logit
Response: Cucop
Terms added sequentially (first to last)

     Df  Deviance Resid.  Df   Resid. Dev   Pr(>Chi)
NULL                      47       64.443
Cu    1    5.7541         46       58.689    0.01645 *
```

```
> odd.ratio = exp(coef(analise));odd.ratio
(Intercept)      CuCut
  1.1538462   0.2166667
> ICbeta1=confint.default(analise,level=0.95);ICbeta1
                 2.5 %     97.5 %
(Intercept) -0.599593  0.8857946
CuCut       -2.853045 -0.2057452
> ICOR1=exp(ICbeta1);ICOR1
                  2.5 %       97.5 %
(Intercept) 0.54903507   2.4249106
CuCut       0.05766844   0.8140405
```

Signals

FS-1a

```
> analise=glm(FS1a~trat, family=binomial)
> summary(analise)

Call:
glm(formula = FS1a ~ trat, family = binomial)

Deviance Residuals:
   Min      1Q  Median      3Q     Max
-1.046  -0.999  -0.999   1.315   1.367

Coefficients:
            Estimate Std. Error z value Pr(>|z|)
(Intercept)  -0.4353     0.3870  -1.125    0.261
tratT         0.1169     0.6047   0.193    0.847

(Dispersion parameter for binomial family taken to be 1)

    Null deviance: 63.422  on 46  degrees of freedom
Residual deviance: 63.384  on 45  degrees of freedom
AIC: 67.384

Number of Fisher Scoring iterations: 4

> anova(analise, test="Chi")
Analysis of Deviance Table

Model: binomial, link: logit
Response: FS1a
Terms added sequentially (first to last)


     Df  Deviance Resid. Df   Resid. Dev  Pr(>Chi)
NULL                     46     63.422
trat  1  0.037317        45     63.384      0.8468
> odd.ratio = exp(coef(analise));odd.ratio
(Intercept)      tratT
  0.6470588   1.1239669
> odd.ratio = exp(coef(analise));odd.ratio
(Intercept)      tratT
  0.6470588   1.1239669
> ICbeta1=confint.default(analise,level=0.95);ICbeta1
                 2.5 %      97.5 %
(Intercept) -1.193732   0.3230959
tratT       -1.068293   1.3020215
> ICOR1=exp(ICbeta1);ICOR1
                 2.5 %       97.5 %
(Intercept) 0.3030880   1.381398
```

```
tratT         0.3435946   3.676722
```

FS-1b

```
> analise=glm(FS1b~trat, family=binomial)
> summary(analise)

Call:
glm(formula = FS1b ~ trat, family = binomial)

Deviance Residuals:
    Min       1Q   Median       3Q      Max
-1.1173  -1.1173  -0.5863   1.2388   1.9214

Coefficients:
             Estimate  Std. Error  z value Pr(>|z|)
(Intercept)  -0.1431     0.3789     -0.378   0.7057
tratT        -1.5309     0.7344     -2.084   0.0371 *
(Dispersion parameter for binomial family taken to be 1)

    Null deviance: 60.284  on 46  degrees of freedom
Residual deviance: 55.247  on 45  degrees of freedom
AIC: 59.247

Number of Fisher Scoring iterations: 3

> anova(analise, test="Chi")
Analysis of Deviance Table

Model: binomial, link: logit
Response: FS1b
Terms added sequentially (first to last)

             Df Deviance Resid.   Df Resid. Dev Pr(>Chi)
NULL                              46     60.284
trat          1      5.0364       45     55.247      0.02482 *

> odd.ratio = exp(coef(analise));odd.ratio
(Intercept)       tratT
  0.8666667   0.2163462
> ICbeta1=confint.default(analise,level=0.95);ICbeta1
               2.5 %      97.5 %
(Intercept) -0.8857936  0.59959195
tratT       -2.9703667 -0.09138453
> ICOR1=exp(ICbeta1);ICOR1
                  2.5 %       97.5 %
(Intercept) 0.4123868   1.8213754
tratT         0.0512845   0.9126667
```

MS-1

```
> analise=glm(MS1~trat, family=binomial)
> summary(analise)

Call:
glm(formula = MS1 ~ trat, family = binomial)

Deviance Residuals:
    Min       1Q   Median       3Q      Max
-0.8806  -0.8806  -0.4717  -0.4717   2.1219

Coefficients:
```

	Estimate	Std. Error	z value	Pr(>|z|)
(Intercept)	-0.7472	0.4047	-1.847	0.0648 .
tratT	-1.3929	0.8500	-1.639	0.1013

(Dispersion parameter for binomial family taken to be 1)

Null deviance: 51.147 on 46 degrees of freedom
Residual deviance: 47.952 on 45 degrees of freedom
AIC: 51.952
Number of Fisher Scoring iterations: 4

> anova(analise, test="Chi")
Analysis of Deviance Table

Model: binomial, link: logit
Response: MS1
Terms added sequentially (first to last)

	Df	Deviance	Resid. Df	Resid. Dev	Pr(>Chi)
NULL			46	51.147	
trat	1	3.1952	45	47.952	0.07385 .

> odd.ratio = exp(coef(analise));odd.ratio
(Intercept) tratT
0.4736842 0.2483660
> ICbeta1=confint.default(analise,level=0.95);ICbeta1

	2.5 %	97.5 %
(Intercept)	-1.540316	0.04588739
tratT	-3.058837	0.27313340

> ICOR1=exp(ICbeta1);ICOR1

	2.5 %	97.5 %
(Intercept)	0.21431333	1.046957
tratT	0.04694226	1.314076

Chinavia impicticornis

Proportion of response

> analise=glm(Ciresp~Ci, family=binomial)
> summary(analise)

Call:
glm(formula = Ciresp ~ Ci, family = binomial)
Deviance Residuals:

Min	1Q	Median	3Q	Max
-1.706	-1.144	0.729	0.729	1.212

Coefficients:

	Estimate	Std. Error	z value	Pr(>|z|)
(Intercept)	1.1896	0.4317	2.756	0.00585 **
CiCit	-1.2696	0.5887	-2.157	0.03104 *

Null deviance: 72.103 on 54 degrees of freedom
Residual deviance: 67.214 on 53 degrees of freedom
AIC: 71.214
Number of Fisher Scoring iterations: 4

> anova(analise, test="Chi")
Analysis of Deviance Table
Model: binomial, link: logit
Response: Ciresp
Terms added sequentially (first to last)

```
            Df Deviance Resid. Df  Resid. Dev Pr(>Chi)
NULL                          54   72.103
Ci          1      4.8893     53   67.214     0.02702 *

> odd.ratio = exp(coef(analise))
> odd.ratio
(Intercept)      CiCit
  3.2857143   0.2809365
> ICbeta1=confint.default(analise,level=0.95);ICbeta1
              2.5 %     97.5 %
(Intercept)  0.3435352  2.0356329
CiCit       -2.4234973 -0.1157563
> ICOR1=exp(ICbeta1);ICOR1
                  2.5 %        97.5 %
(Intercept) 1.40992315    7.6570970
CiCit        0.08861118   0.8906923
```

Female

Latency

```
> dados=read.table("tlcif.txt", h=T)
> attach(dados)
> names(dados)
[1] "Female" "TL"
> shapiro.test(TL)

        Shapiro-Wilk normality test
data:  TL
W = 0.87333, p-value = 0.000818

> analise=glm(TL~Female, family=quasipoisson)
> summary(analise)

Call:
glm(formula = TL ~ Female, family = quasipoisson)

Deviance Residuals:
    Min       1Q   Median      3Q      Max
-13.587   -7.586   -2.496   4.421   15.627

Coefficients:
                     Estimate Std. Error t value Pr(>|t|)
(Intercept)           4.6266     0.1748  26.475   <2e-16 ***
FemaleTreatment  -0.4179     0.3630  -1.151    0.258

(Dispersion parameter for quasipoisson family taken to be 74.88364)
    Null deviance: 2580.7  on 34  degrees of freedom
Residual deviance: 2474.2  on 33  degrees of freedom
AIC: NA
Number of Fisher Scoring iterations: 5
```

Response time

```
> dados=read.table("tccif.txt", h=T)
> shapiro.test(RT)

        Shapiro-Wilk normality test
data:  RT
W = 0.91317, p-value = 0.01363

> analise=glm(RT~Female, family=quasipoisson)
```

```
> summary(analise)

Call:
glm(formula = RT ~ Female, family = quasipoisson)

Deviance Residuals:
    Min       1Q   Median       3Q      Max
-19.430   -1.493    1.672    4.176   15.238

Coefficients:
                Estimate Std. Error t value Pr(>|t|)
(Intercept)      6.66158    0.05491 121.315   <2e-16 ***
FemaleTreatment -0.02490    0.10448  -0.238    0.813

(Dispersion parameter for quasipoisson family taken to be 54.21708)
    Null deviance: 1812.0  on 31  degrees of freedom
Residual deviance: 1808.9  on 30  degrees of freedom
AIC: NA
Number of Fisher Scoring iterations: 4
```

Male

Latency

```
> dados=read.table("tlcim.txt", h=T)
> shapiro.test(TL)

        Shapiro-Wilk normality test
data:  TL
W = 0.83963, p-value = 0.0003071

> analise=glm(TL~Machos, family=quasipoisson)
> summary(analise)

Call:
glm(formula = TL ~ Machos, family = quasipoisson)

Deviance Residuals:
    Min       1Q   Median       3Q      Max
-12.697   -8.604   -5.761    5.499   20.653



Coefficients:
                 Estimate Std. Error t value Pr(>|t|)
(Intercept)       4.474      0.222  20.155   <2e-16 ***
MachosTreatment   0.196      0.385   0.509    0.615
(Dispersion parameter for quasipoisson family taken to be 95.03718)
    Null deviance: 2636.1  on 30  degrees of freedom
Residual deviance: 2612.0  on 29  degrees of freedom
AIC: NA
Number of Fisher Scoring iterations: 5
```

Response Time

```
> dados=read.table("tccim.txt", h=T)
> attach(dados)
> names(dados)
[1] "Male" "RT"
> shapiro.test(RT)

        Shapiro-Wilk normality test
data:  RT
```

```
aW = 0.93531, p-value = 0.06126

> analise=glm(RT~Male, family=quasipoisson)
> summary(analise)

Call:
glm(formula = RT ~ Male, family = quasipoisson)

Deviance Residuals:
    Min       1Q   Median       3Q      Max
-28.828   -7.623   -1.617    5.665   18.187

Coefficients:
              Estimate Std. Error t value Pr(>|t|)
(Intercept)    6.71913    0.07648  87.853   <2e-16 ***
MaleTreatment -0.37910    0.14624  -2.592   0.0148 *

(Dispersion parameter for quasipoisson family taken to be 96.87755)
    Null deviance: 3776.9  on 30  degrees of freedom
Residual deviance: 3090.5  on 29  degrees of freedom
AIC: NA
Number of Fisher Scoring iterations: 5
```

Proportion of couple formation

```
> res = prop.test(x = c(12, 7), n = c(23, 12))
> res
        2-sample test for equality of proportions with continuity correction

data:  c(12, 7) out of c(23, 12)
X-squared = 8.1683e-31, df = 1, p-value = 1
alternative hypothesis: two.sided
95 percent confidence interval:
 -0.4688521  0.3456637
sample estimates:
   prop 1    prop 2
0.5217391 0.5833333
```

Proportion of copulation

```
> analise=glm(Cicop~Ci, family=binomial)
> summary(analise)

Call:
glm(formula = Cicop ~ Ci, family = binomial)

Deviance Residuals:
    Min       1Q   Median       3Q      Max
-1.0108  -1.0108  -0.8106   1.3537   1.5956

Coefficients:
            Estimate  Std. Error z value  Pr(>|z|)
(Intercept)  -0.4055    0.3727    -1.088   0.277
CiCit        -0.5390    0.5808    -0.928   0.353

(Dispersion parameter for binomial family taken to be 1)
    Null deviance: 70.905  on 54  degrees of freedom
Residual deviance: 70.028  on 53  degrees of freedom
AIC: 74.028

Number of Fisher Scoring iterations: 4

> anova(analise, test="Chi")
```

```
Analysis of Deviance Table
Model: binomial, link: logit
Response: Cicop
Terms added sequentially (first to last)


             Df  Deviance Resid. Df    Resid. Dev Pr(>Chi)
NULL                             54     70.905
Ci            1      0.87624     53     70.028     0.3492

> odd.ratio = exp(coef(analise));odd.ratio
(Intercept)      CiCit
  0.6666667   0.5833333
> ICbeta1=confint.default(analise,level=0.95);ICbeta1
              2.5 %    97.5 %
(Intercept) -1.135901 0.3249703
CiCit       -1.677297 0.5993045
> ICOR1=exp(ICbeta1);ICOR1
                 2.5 %      97.5 %
(Intercept) 0.3211328    1.383990
CiCit        0.1868783   1.820852
```

Signals

FS-1a

```
> analise=glm(FS1a~trat, family=binomial)
> summary(analise)

Call:
glm(formula = FS1a ~ trat, family = binomial)

Deviance Residuals:
    Min       1Q   Median       3Q      Max
-1.0108  -1.0108  -0.5056   1.3537   2.0593

Coefficients:
             Estimate  Std. Error z value   Pr(>|z|)
(Intercept)  -0.4055     0.3727    -1.088   0.2766
tratT         -1.5870     0.7195    -2.206   0.0274 *
(Dispersion parameter for binomial family taken to be 1)

    Null deviance: 64.455  on 54  degrees of freedom
Residual deviance: 58.727  on 53  degrees of freedom
AIC: 62.727
Number of Fisher Scoring iterations: 4

> anova(analise, test="Chi")
Analysis of Deviance Table

Model: binomial, link: logit
Response: FS1a
Terms added sequentially (first to last)

             Df Deviance Resid.  Df  Resid. Dev Pr(>Chi)
NULL                             54    64.455
trat          1     5.7278       53    58.727      0.0167 *

> odd.ratio = exp(coef(analise));odd.ratio
(Intercept)      tratT
  0.6666667   0.2045455
> ICbeta1=confint.default(analise,level=0.95);ICbeta1
```

```
               2.5 %     97.5 %
(Intercept) -1.135901  0.3249703
tratT       -2.997146 -0.1767839
> ICOR1=exp(ICbeta1);ICOR1
                     2.5 %        97.5 %
(Intercept) 0.32113279   1.3839896
tratT          0.04992935   0.8379609
```

FS-1b

```
> analise=glm(FS1b~trat, family=binomial)
> summary(analise)

Call:
glm(formula = FS1b ~ trat, family = binomial)
Deviance Residuals:
   Min      1Q  Median      3Q     Max
-1.066  -1.066  -1.011   1.293   1.354

Coefficients:
               Estimate Std. Error   z value  Pr(>|z|)
(Intercept)  -0.2683     0.3684    -0.728     0.467
tratT          -0.1372     0.5499    -0.249     0.803
(Dispersion parameter for binomial family taken to be 1)
    Null deviance: 74.767  on 54  degrees of freedom
Residual deviance: 74.704  on 53  degrees of freedom
AIC: 78.704

Number of Fisher Scoring iterations: 4

> anova(analise, test="Chi")
Analysis of Deviance Table

Model: binomial, link: logit
Response: FS1b
Terms added sequentially (first to last)


                Df Deviance Resid.  Df   Resid. Dev Pr(>Chi)
NULL                                  54     74.767
trat            1         0.062329       53     74.704        0.8029
> odd.ratio = exp(coef(analise));odd.ratio
(Intercept)       tratT
  0.7647059   0.8717949
> ICbeta1=confint.default(analise,level=0.95);ICbeta1
               2.5 %    97.5 %
(Intercept) -0.9903893 0.4538613
tratT       -1.2150268 0.9406245
> ICOR1=exp(ICbeta1);ICOR1
                    2.5 %        97.5 %
(Intercept) 0.3714321    1.574380
tratT          0.2967021   2.561581
```

MS-1

```
> analise=glm(MS1~trat, family=binomial)
> summary(analise)

Call:
glm(formula = MS1 ~ trat, family = binomial)

Deviance Residuals:
   Min      1Q  Median      3Q     Max
```

```
-1.121  -1.121  -1.011   1.235   1.354

Coefficients:
              Estimate Std. Error z value   Pr(>|z|)
(Intercept)  -0.1335     0.3660    -0.365    0.715
tratT        -0.2719     0.5483    -0.496    0.620

(Dispersion parameter for binomial family taken to be 1)
                   Null deviance: 75.353  on 54  degrees of freedom
Residual deviance: 75.106  on 53  degrees of freedom
AIC: 79.106

Number of Fisher Scoring iterations: 4

> anova(analise, test="Chi")
Analysis of Deviance Table

Model: binomial, link: logit
Response: MS1
Terms added sequentially (first to last)

              Df Deviance Resid. Df  Resid. Dev  Pr(>Chi)
NULL                             54     75.353
trat           1      0.24688    53     75.106       0.6193
> odd.ratio = exp(coef(analise));odd.ratio
(Intercept)       tratT
  0.8750000   0.7619048
> ICbeta1=confint.default(analise,level=0.95);ICbeta1
              2.5 %     97.5 %
(Intercept) -0.8508048 0.5837420
tratT       -1.3465147 0.8026472
> ICOR1=exp(ICbeta1);ICOR1
                2.5 %        97.5 %
(Intercept) 0.4270711    1.792734
tratT        0.2601454   2.231440
```

MS-2

```
> analise=glm(MS2~trat, family=binomial)
> summary(analise)

Call:
glm(formula = MS2 ~ trat, family = binomial)

Deviance Residuals:
    Min       1Q   Median       3Q      Max
-0.7290  -0.7290  -0.5905  -0.5905   1.9145

Coefficients:
               Estimate Std. Error z value   Pr(>|z|)
(Intercept)  -1.1896     0.4317     -2.756   0.00585 **
tratT        -0.4686      0.6957     -0.674  0.50053

(Dispersion parameter for binomial family taken to be 1)
    Null deviance: 55.044  on 54  degrees of freedom
Residual deviance: 54.580  on 53  degrees of freedom
AIC: 58.58
Number of Fisher Scoring iterations: 4

> anova(analise, test="Chi")
Analysis of Deviance Table

Model: binomial, link: logit
```

Response: MS2
Terms added sequentially (first to last)

```
             Df Deviance Resid. Df  Resid. Dev Pr(>Chi)
NULL                            54     55.044
trat          1      0.46441    53     54.580    0.4956
> odd.ratio = exp(coef(analise));odd.ratio
(Intercept)      tratT
  0.3043478   0.6258503
> ICbeta1=confint.default(analise,level=0.95);ICbeta1
               2.5 %     97.5 %
(Intercept) -2.035633 -0.3435352
tratT       -1.832129  0.8948405
> ICOR1=exp(ICbeta1);ICOR1
                2.5 %      97.5 %
(Intercept) 0.1305978   0.7092585
tratT       0.1600725   2.4469455
```
