# Supplementary material for: Inhibitory Copulation Effect of Vibrational Rival Female Signals of Three Stink Bug Species as a Tool for Mating Disruption
Source: Insects. 2021 Feb 18;12(2):177. doi: 10.3390/insects12020177 (PMC7923018; doi:10.3390/insects12020177)

Figure S3

Inhibitory copulation effect of vibrational rival female signals of three stink bug species as a tool for mating disruption

Aline Moreira Dias, Miguel Borges, Maria Carolina Blassioli Moraes, Matheus Lorran Figueira Coelho, Andrej Čokl, Raul Alberto Laumann

Boxplot of variables

Models diagnosis

Abbreviations used in the figures legends

eh = Euschistus heros
cu = Chinavia ubica
ci = Chinavia impicticornis
TRAT = treatment
C = control
T = treatment
FC = female control
FT = female treatment
MT = male treatment
FS1 = female song 1
FS2 = female song 2
MS1 = male song 1
MS2 = male song 2
DP = pulse duration
TR= repetition time
FD or DF = dominant frequency
PTD = pulse train duration
PTRT = pulse train repetition time

Euschistus heros

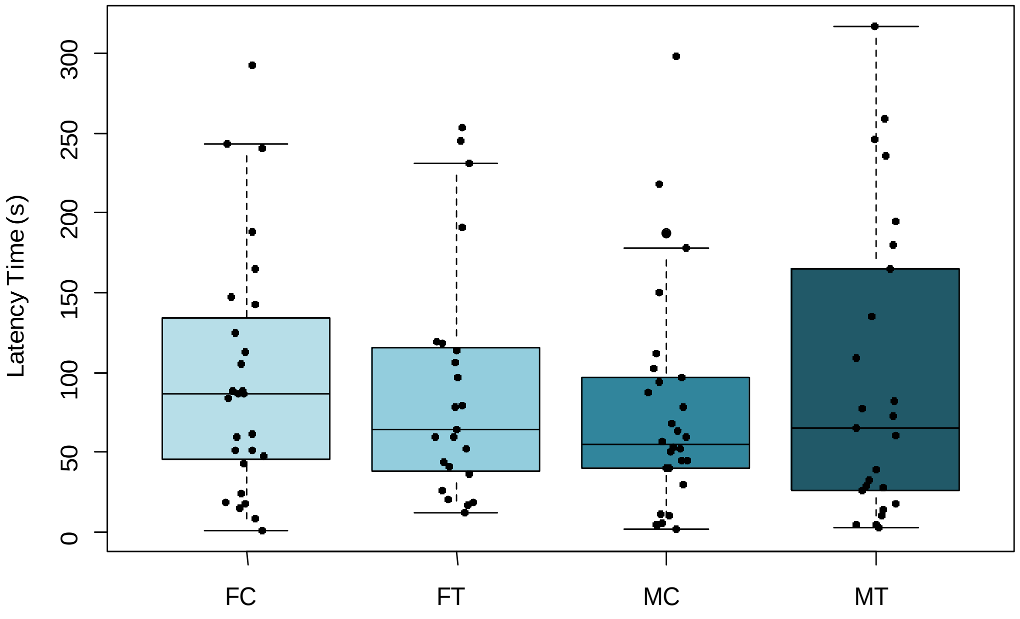

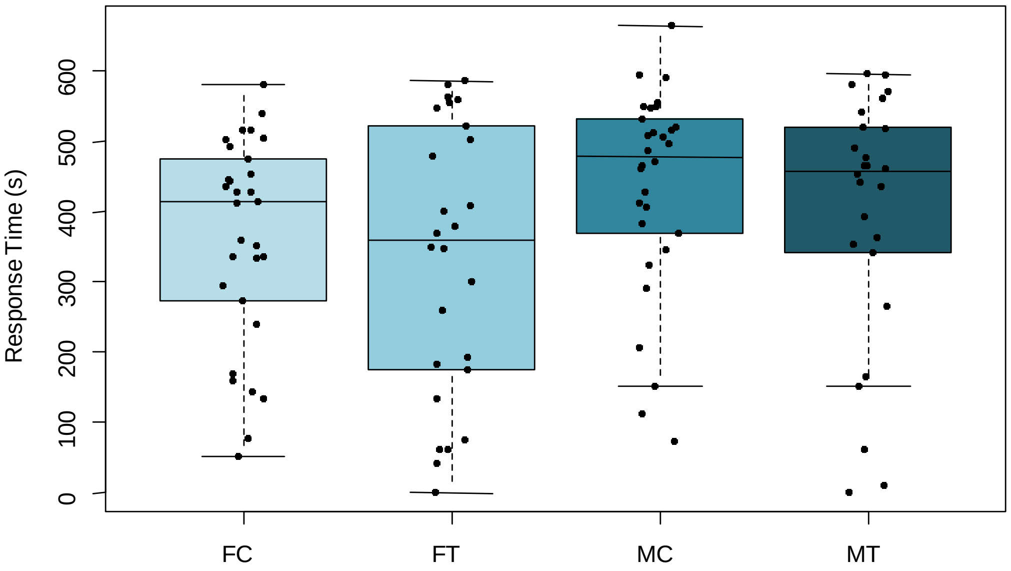

Chinavia ubica

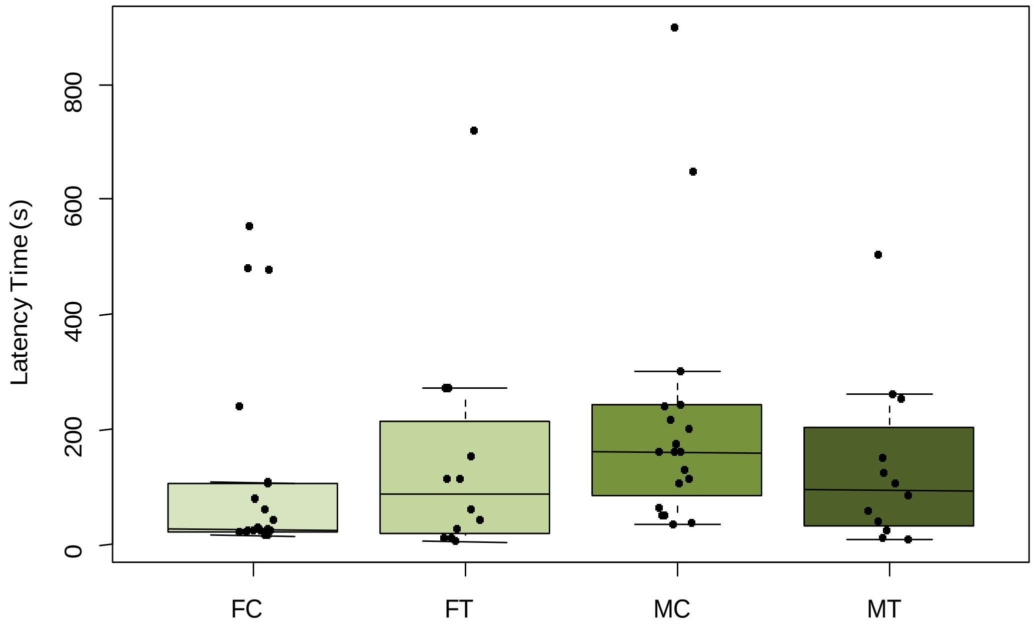

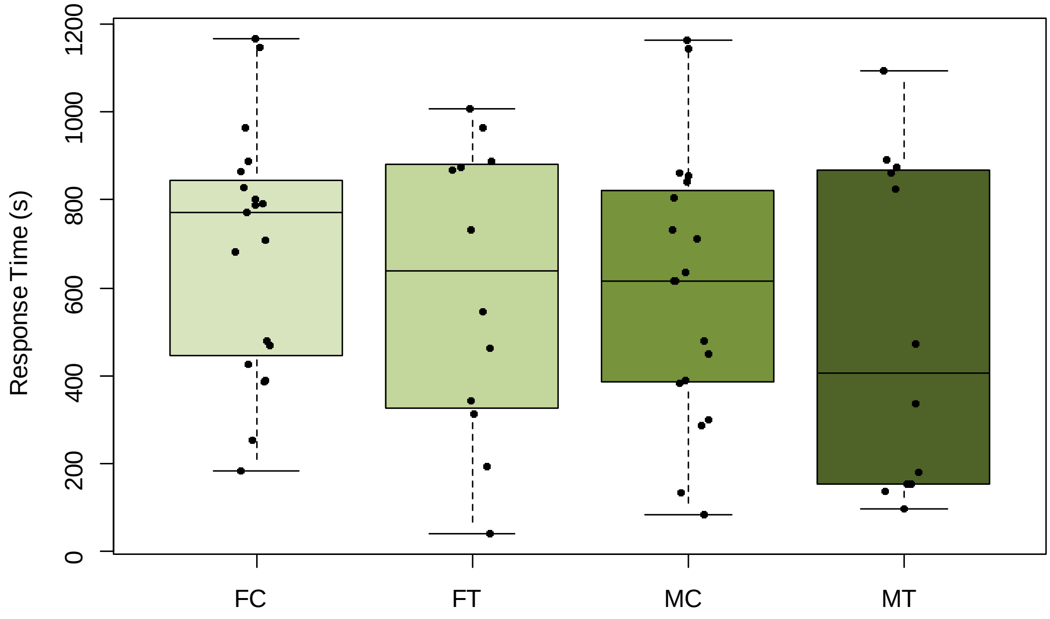

Chinavia impicticornis

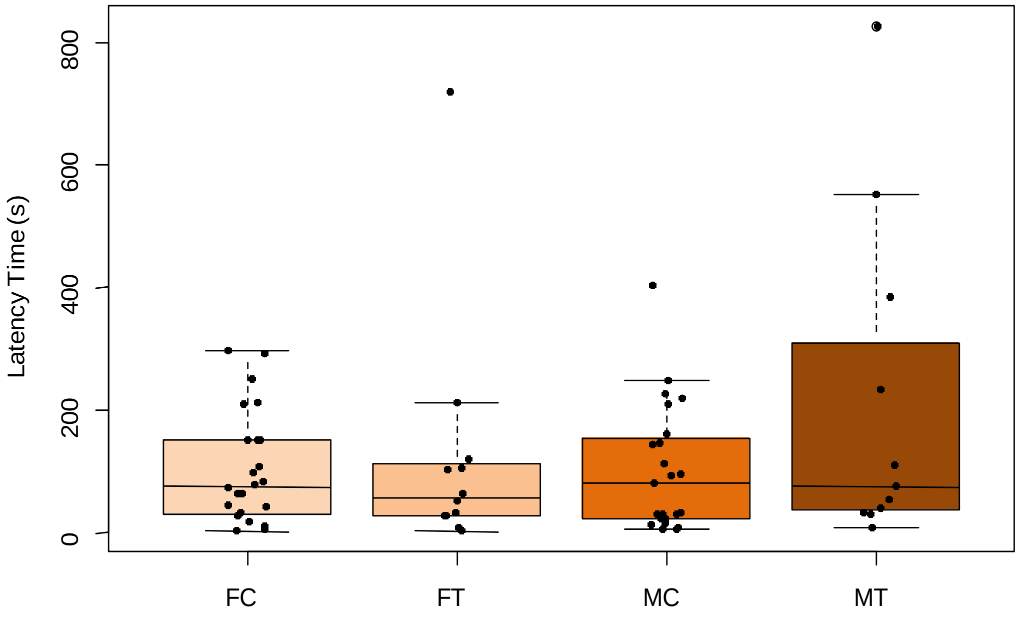

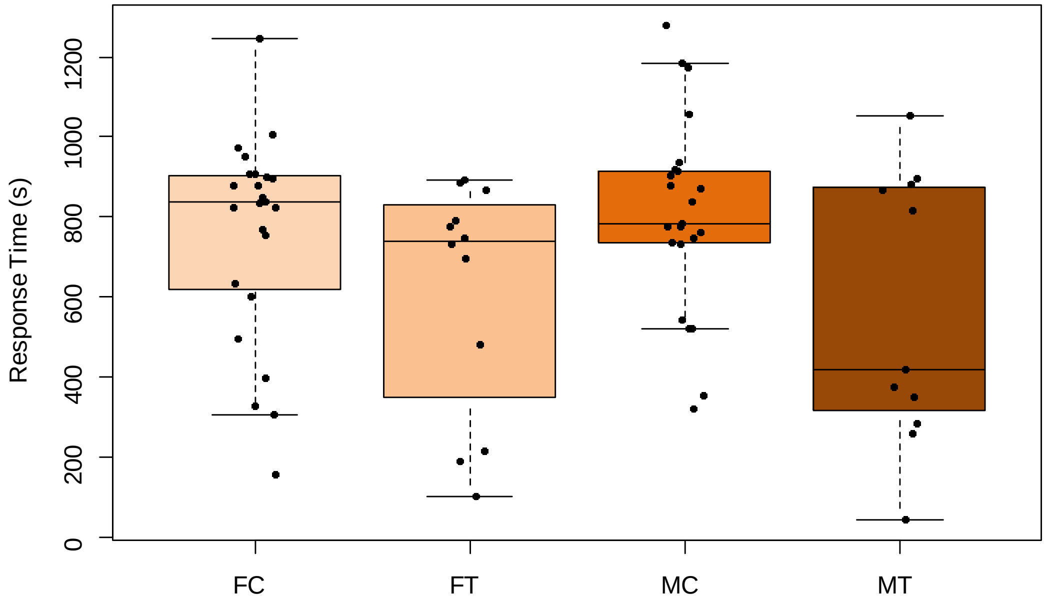

Euschistus heros FS-1

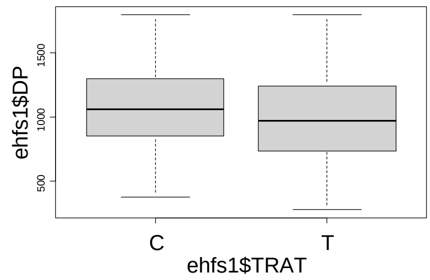

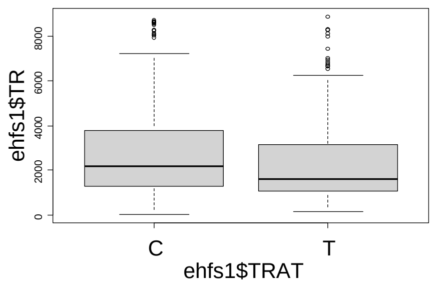

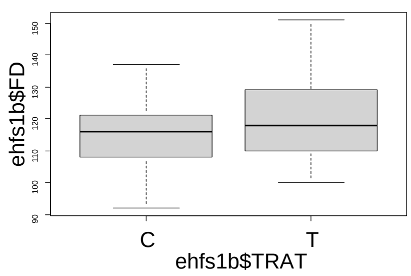

Euschistus heros FS-2

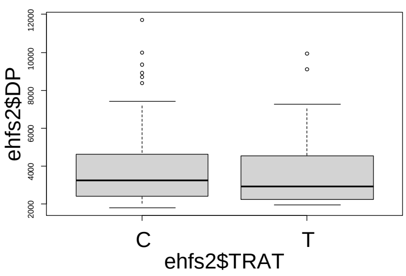

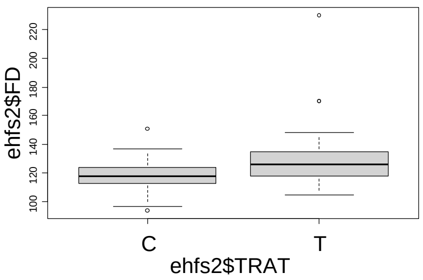

Euschistus heros MS-1

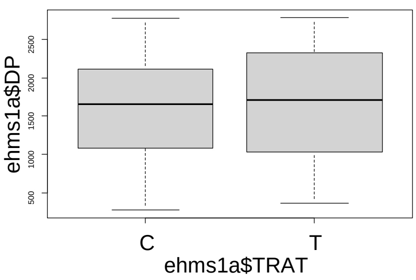

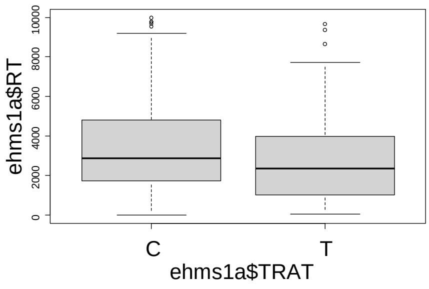

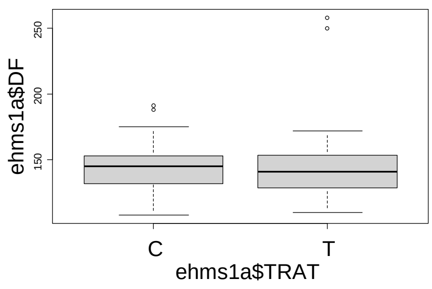

Euschistus heros MS-2

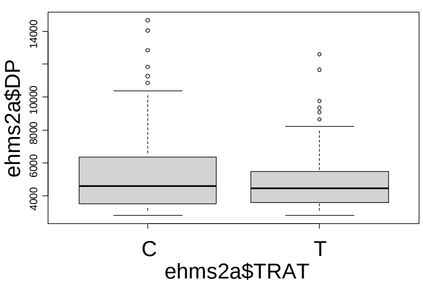

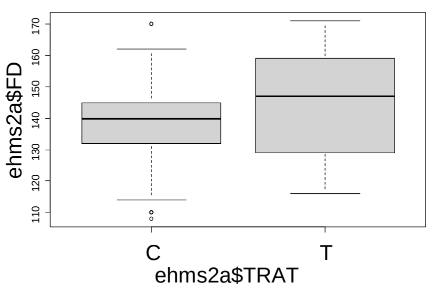

Chinavia ubica FS-1a

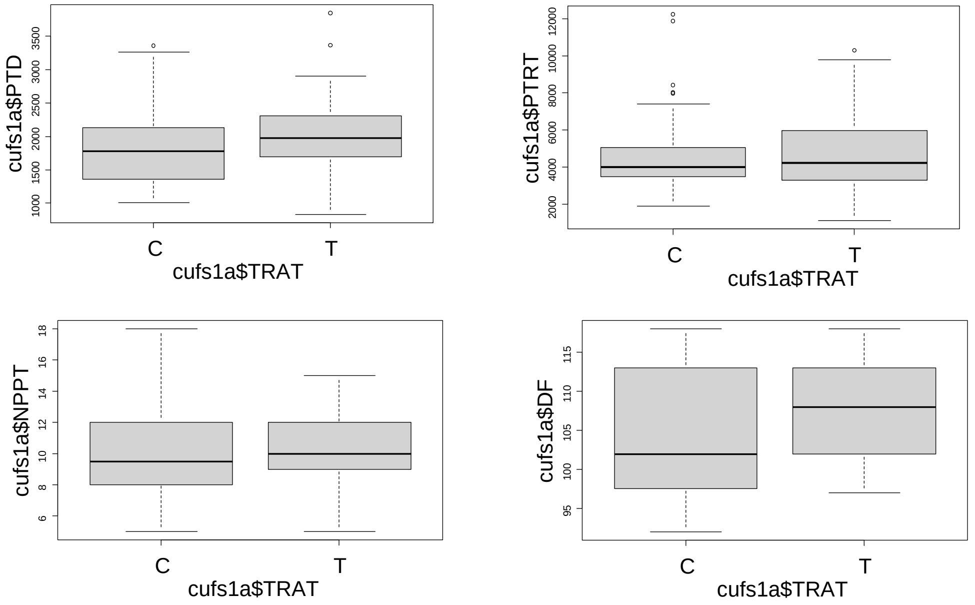

Chinavia ubica FS-1b

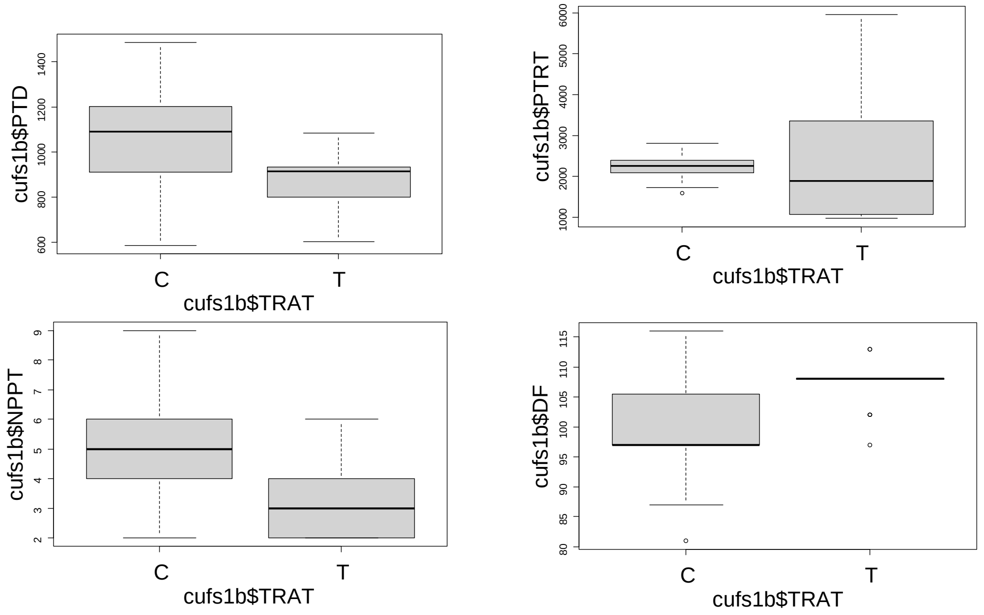

Chinavia ubica MS-1

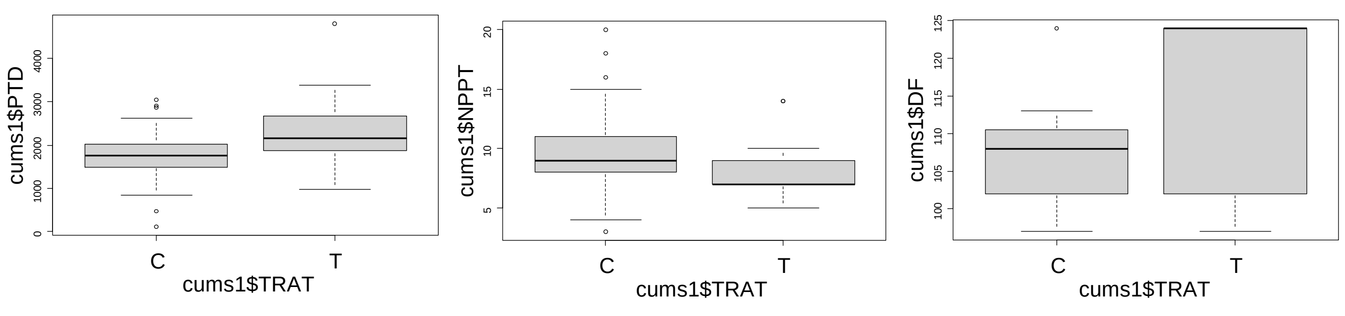

Chinavia impicticornis FS-1a

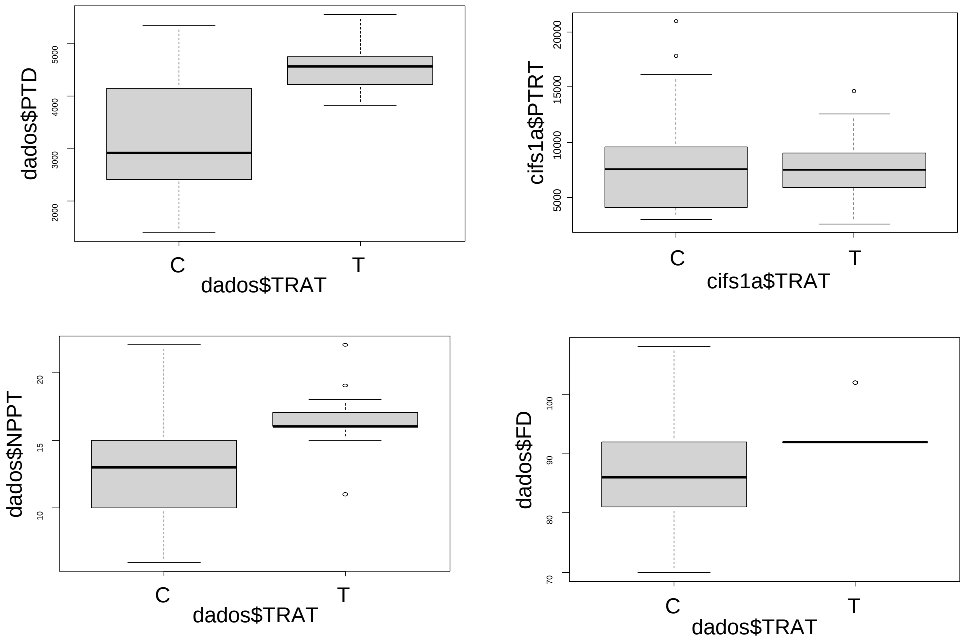

Chinavia impicticornis FS-1b

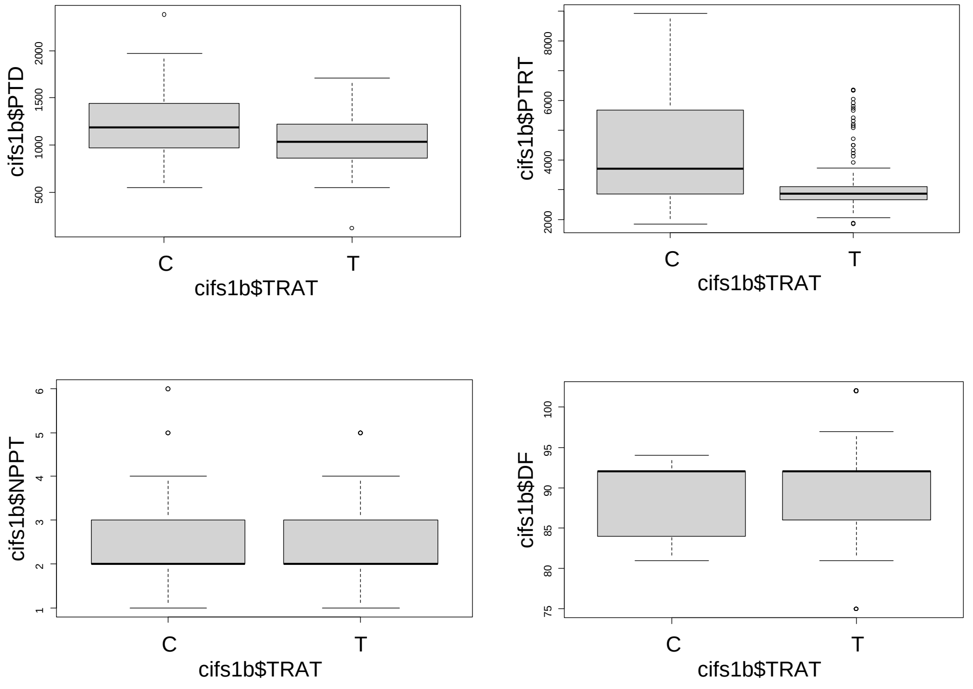

Chinavia impicticornis MS-1

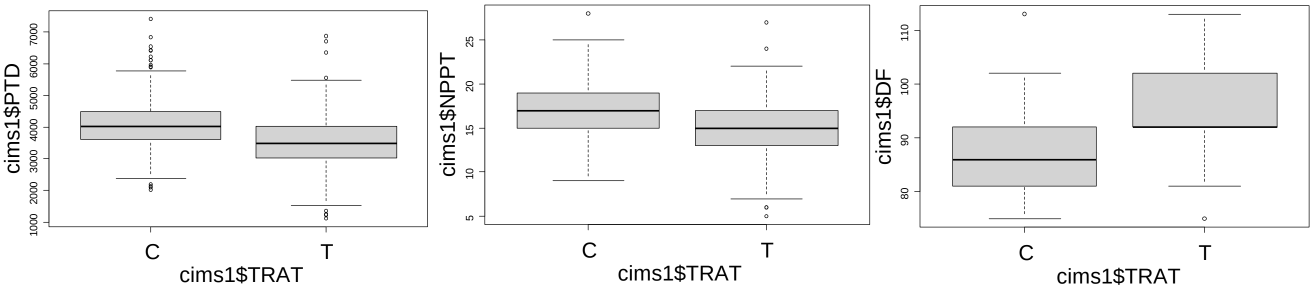

Chinavia impicticornis MS-2

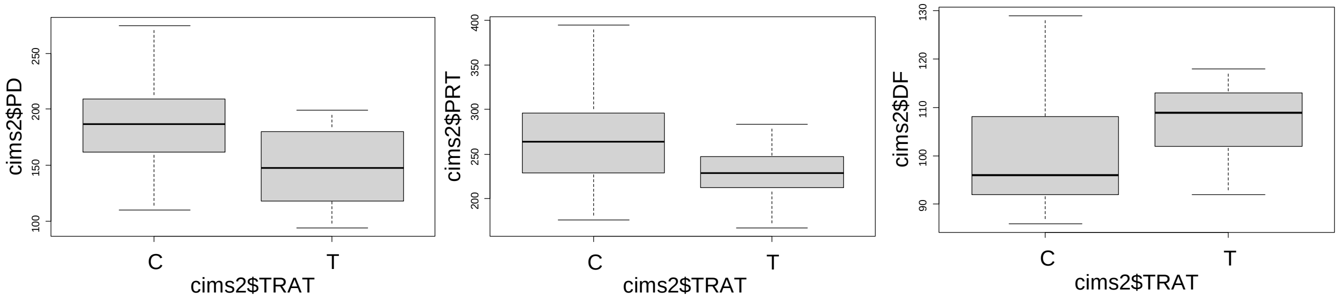

Eushcitus heros

Female

Latency

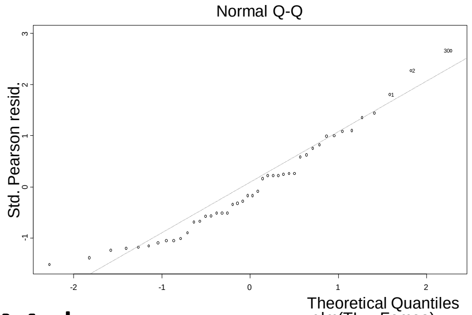

glm(TL ~ Femea)

Response time

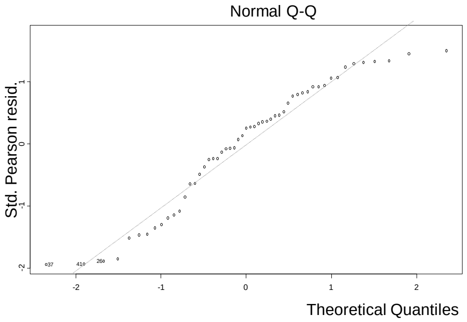

glm(RT ~ Femea)

Male

Latency

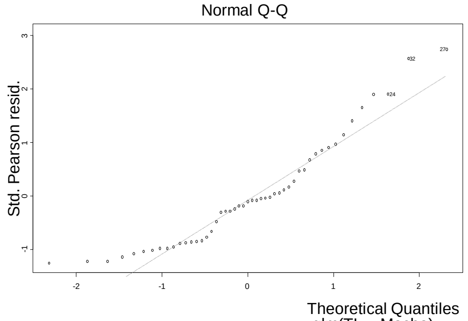

glm(TL ~ Macho)

Response time

glm(RT ~ Femea)

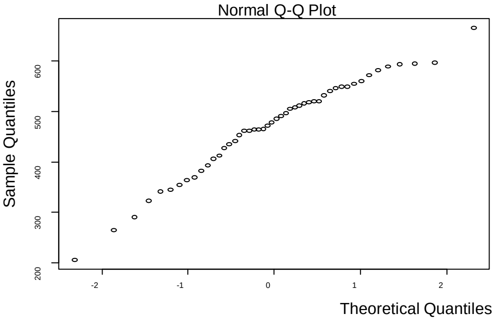

glm(RT ~ Femea)

Chinavia ubica

Female

Latency

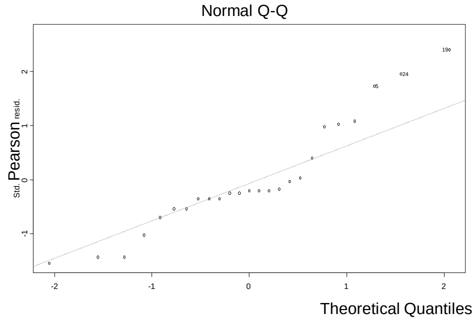

glm(TL ~ Femea)

Response time

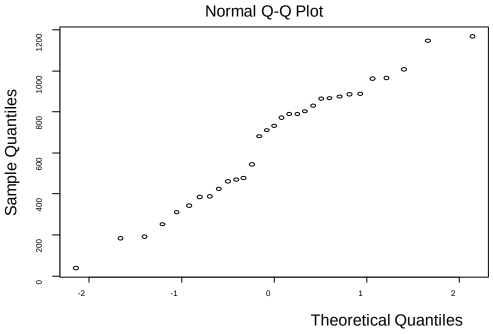

glm(RT ~ Femea)

Male

Latency

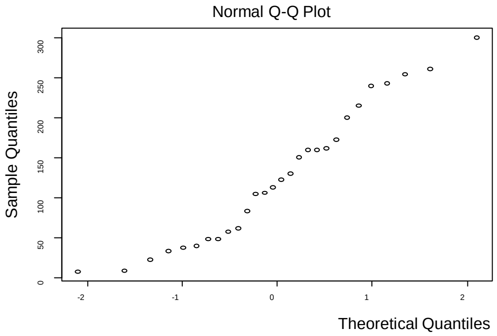

glm(TL ~ Femea)

Response time

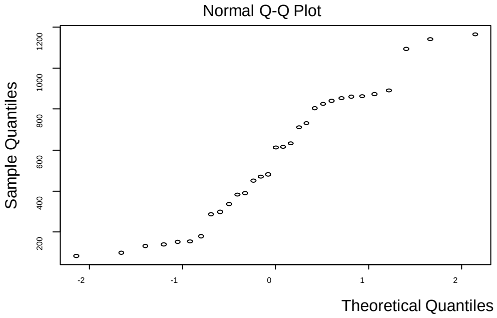

glm(RT ~ Femea)

Chinavia impicticornis

Female

Latency

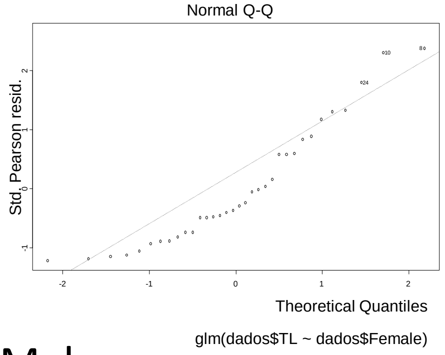

Response time

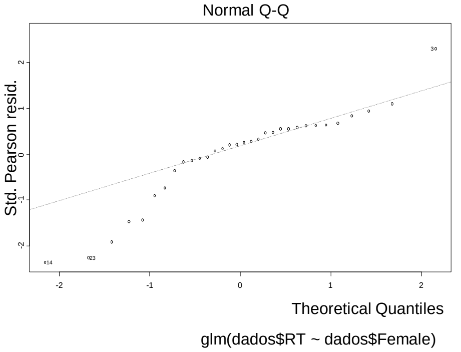

Male

Latency

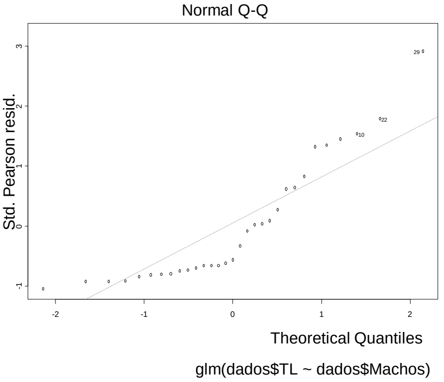

Response time

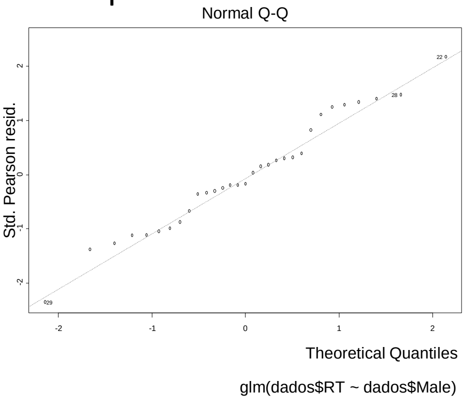

Euschsitus heros – FS-1

Pulse duration

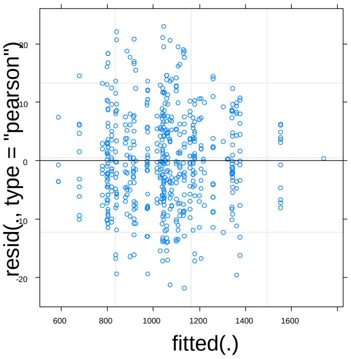

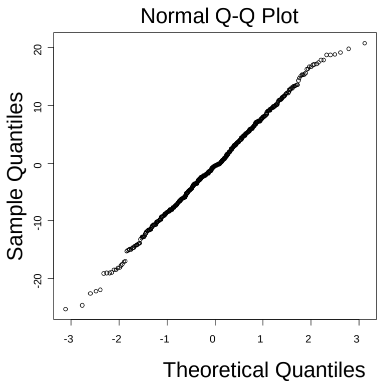

Repetition time

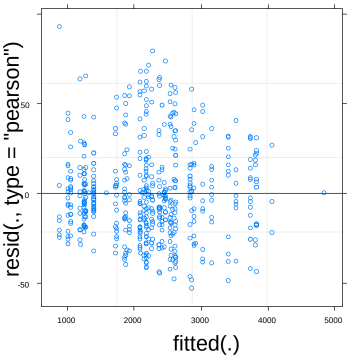

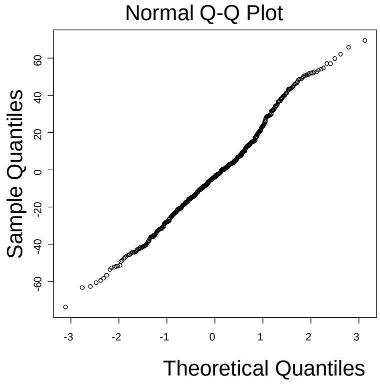

Dominant Frequency

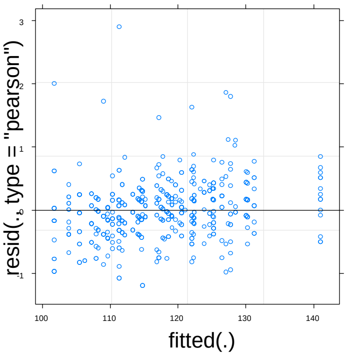

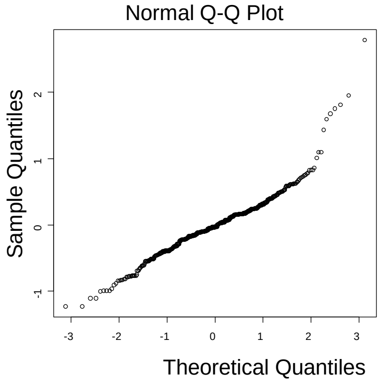

Euschsitus heros – FS-2

Pulse duration

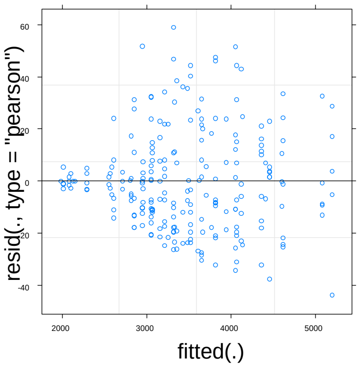

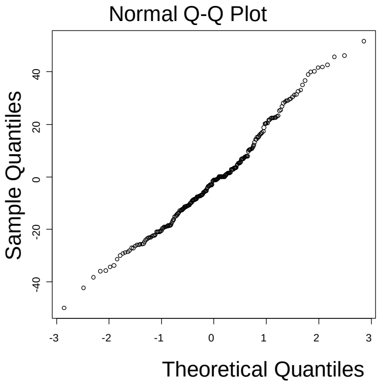

Dominant Frequency

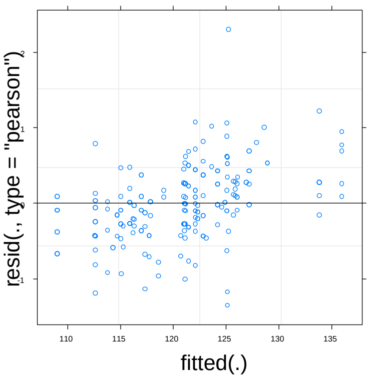

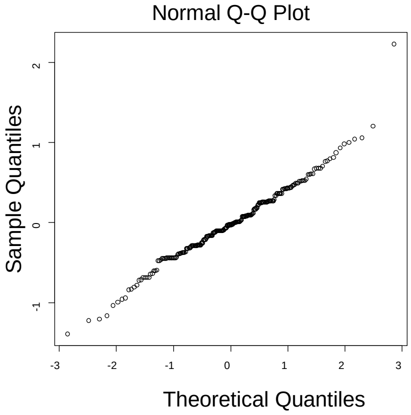

Euschsitus heros – MS-1

Pulse duration

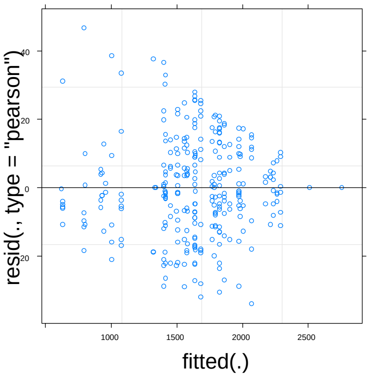

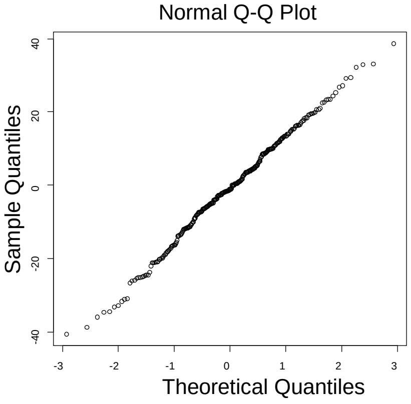

Repetition time

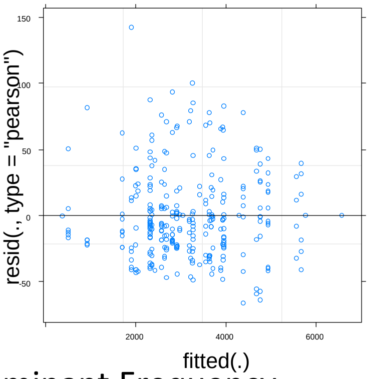

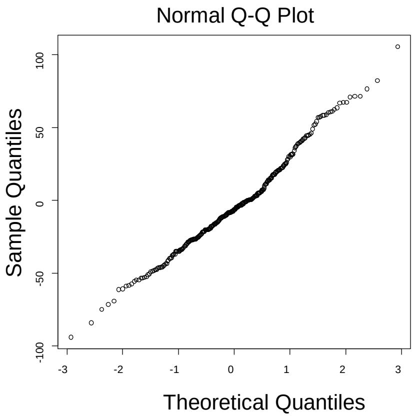

Dominant Frequency

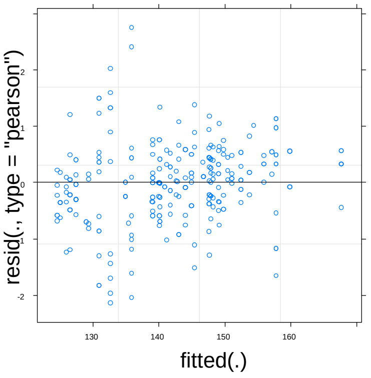

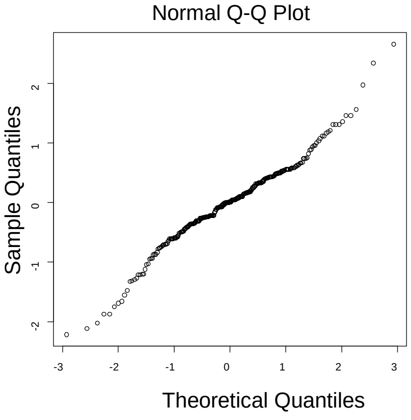

Euschsitus heros – MS-2

PD

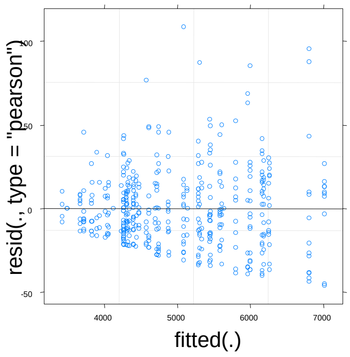

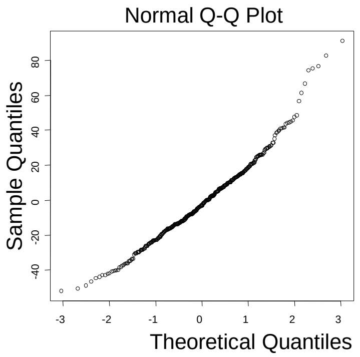

DF

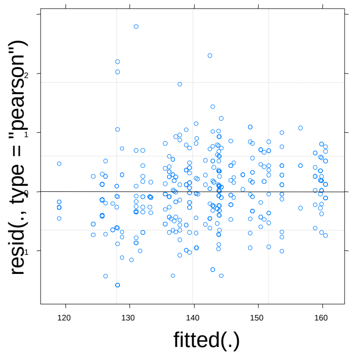

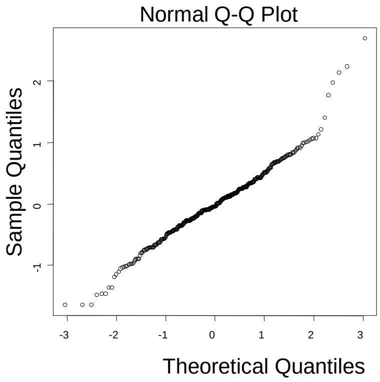

Chinavia ubica – FS-1a

PTD

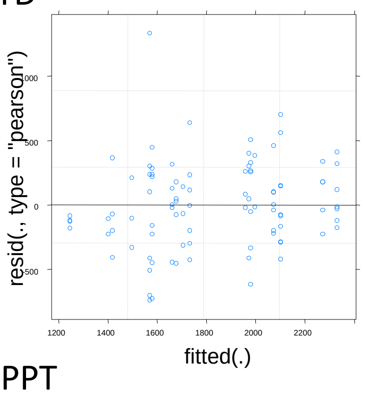

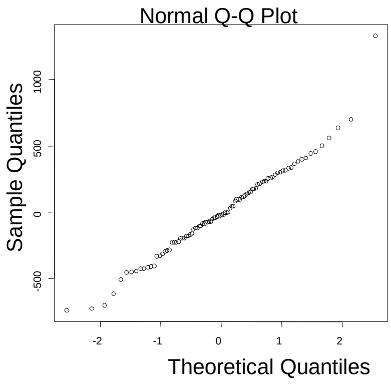

NPPT

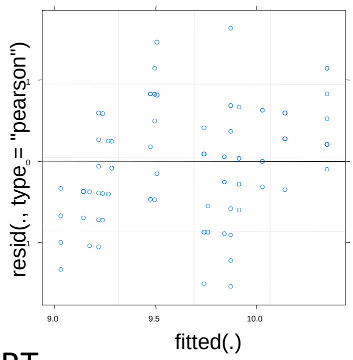

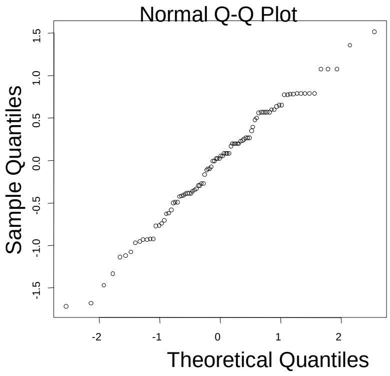

PTRT

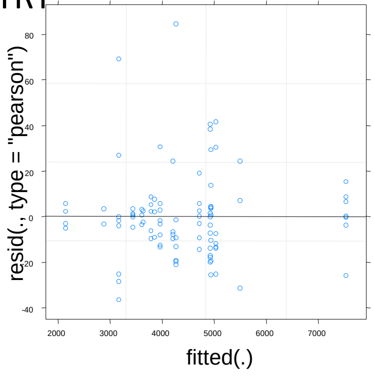

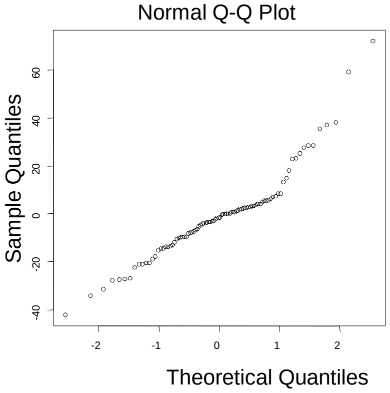

Dominant Frequency

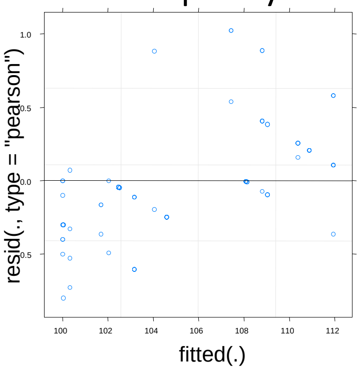

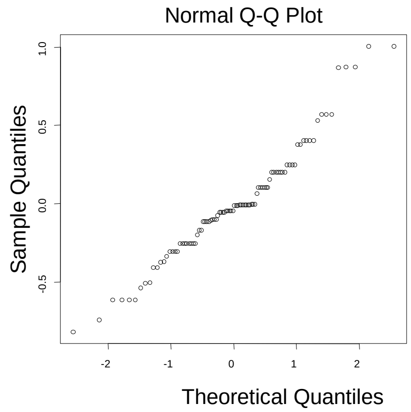

Chinavia ubica – FS-1b

Pulse train duration

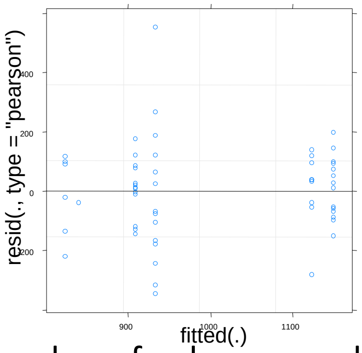

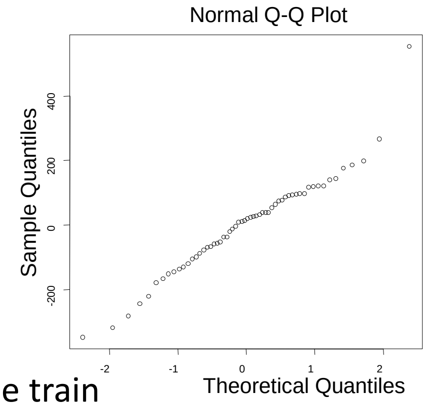

Number of pulses per pulse train

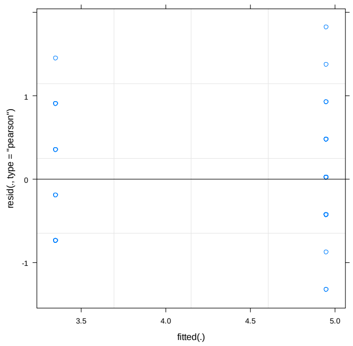

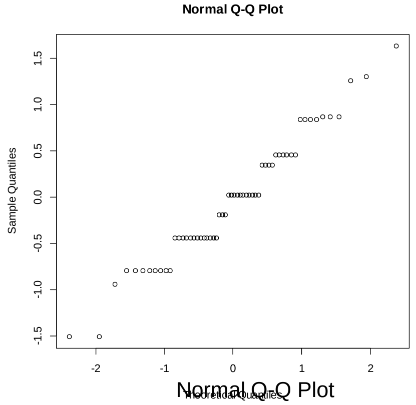

Pulse train repetition time

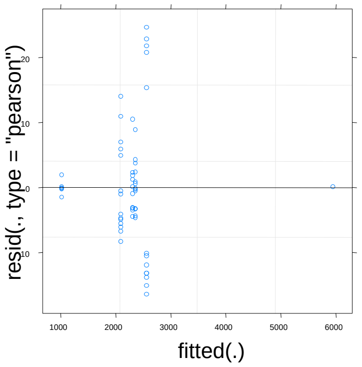

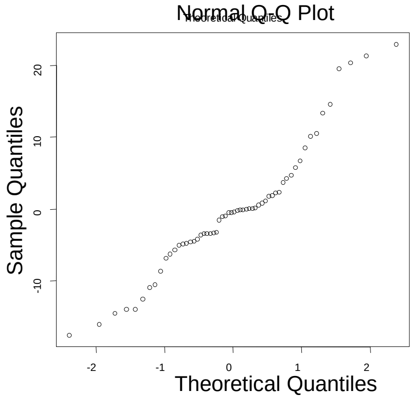

Dominant Frequency

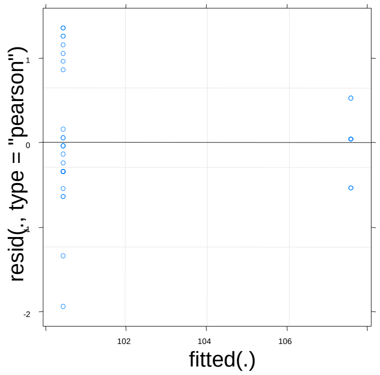

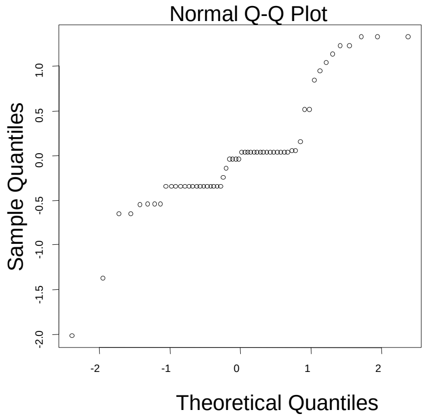

Chinavia ubica – MS-1

Pulse train duration

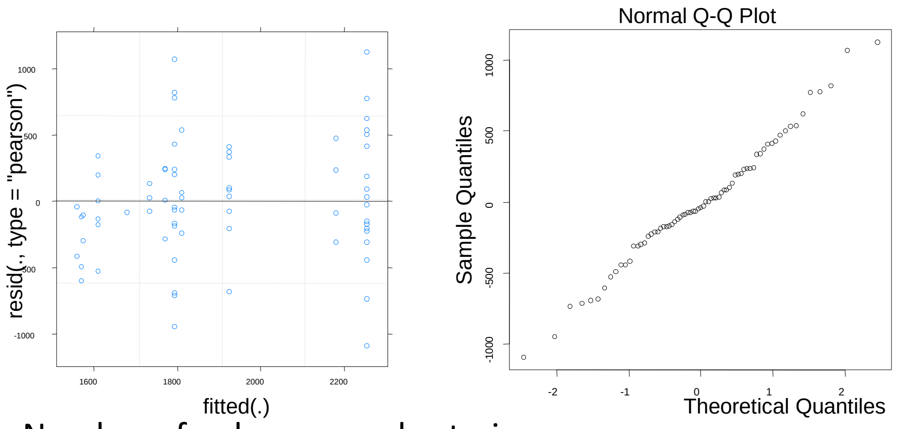

Number of pulses per pulse train

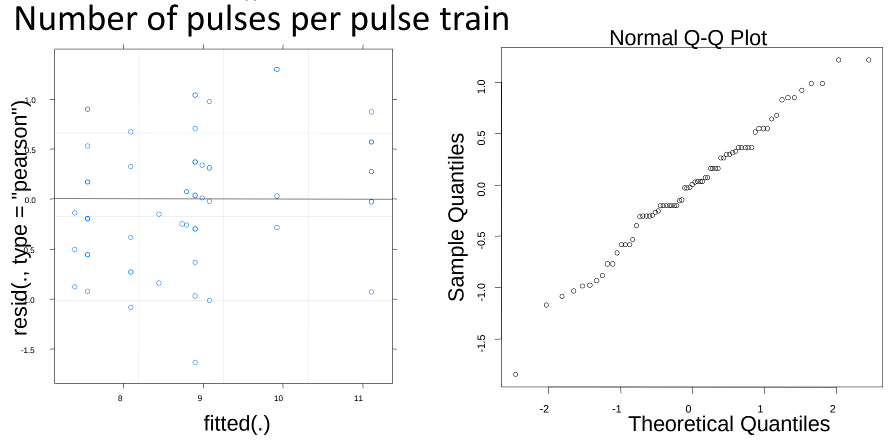

Dominant Frequency

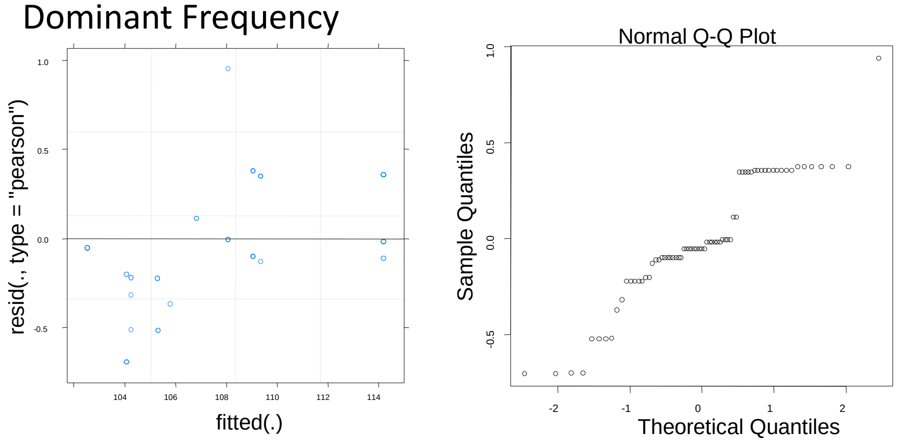

Chinavia impicticornis FS-1a

Pulse train duration

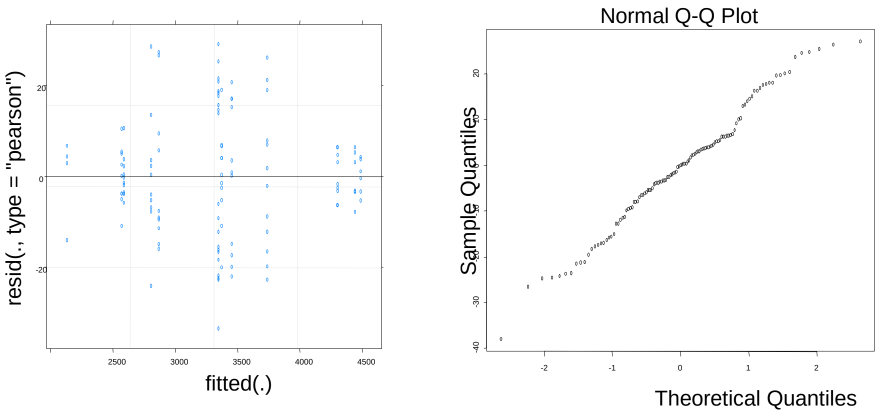

Number of pulses per pulse train

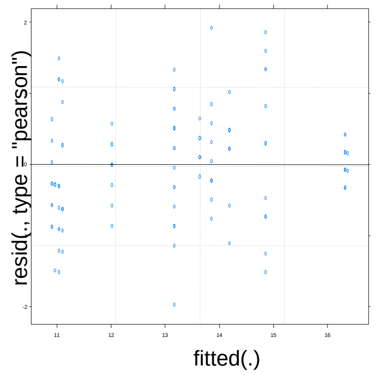

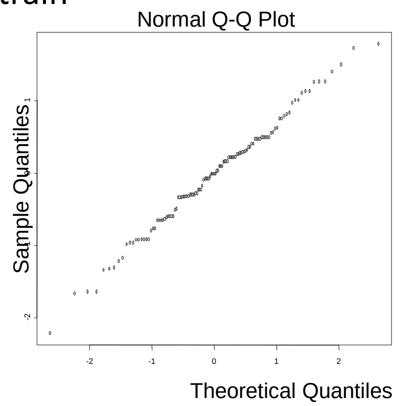

Pulse train repetition time

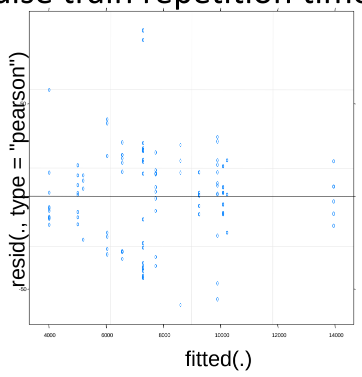

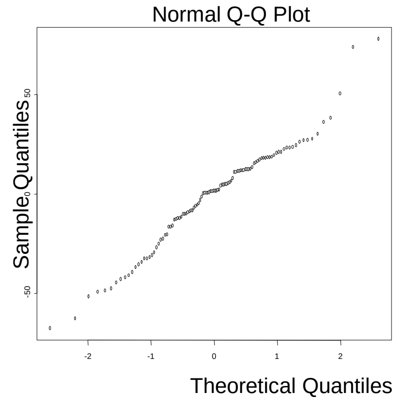

Dominant Frequency

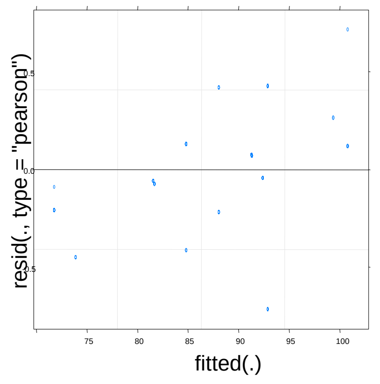

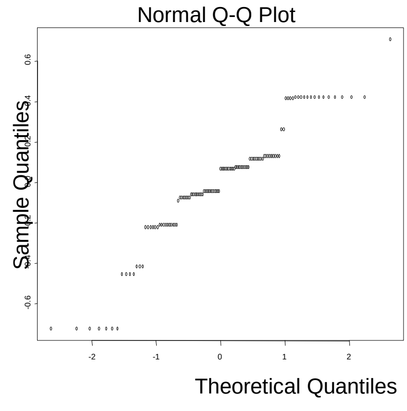

Chinavia impicticornis FS-1b

Pulse train duration

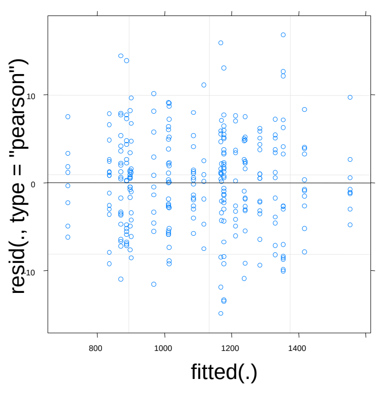

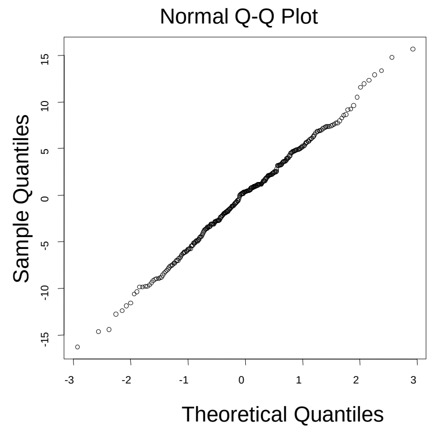

Number of pulses per pulse train

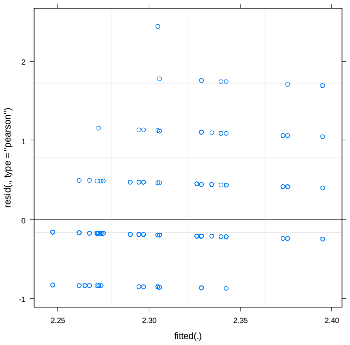

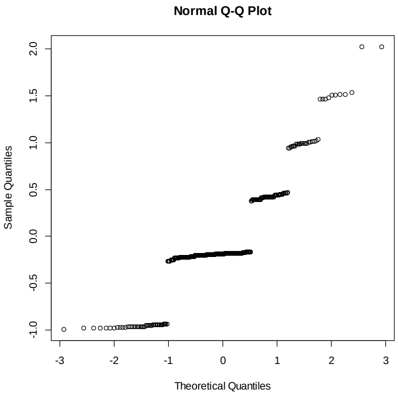

Pulse train repetition time

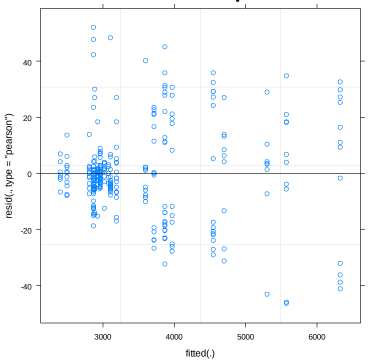

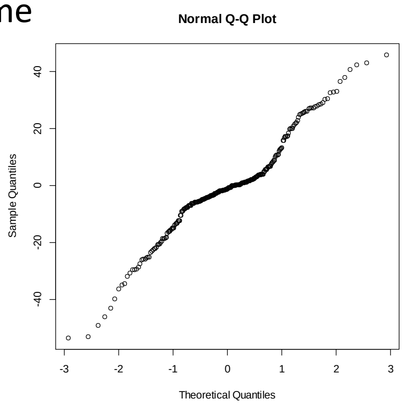

Dominant Frequency

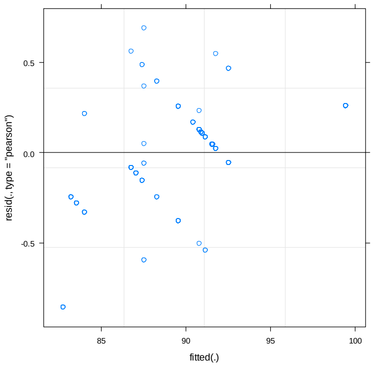

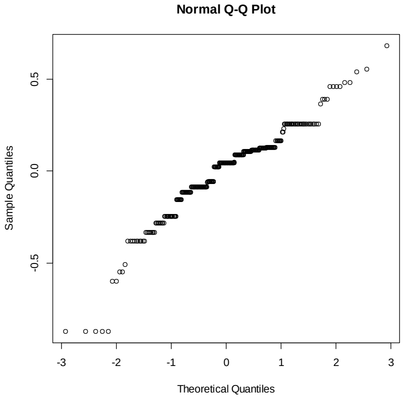

Chinavia impicticornis MS-1

Pulse train duration

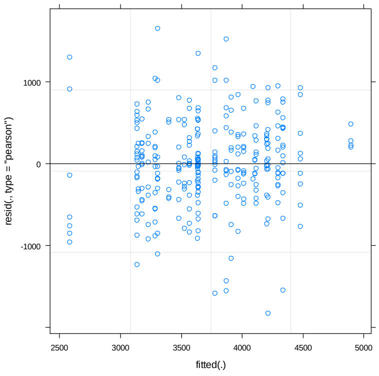

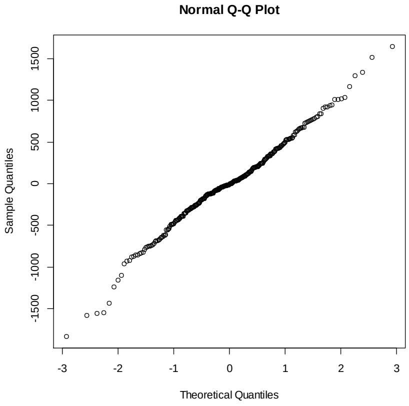

Number of pulses per pulse train

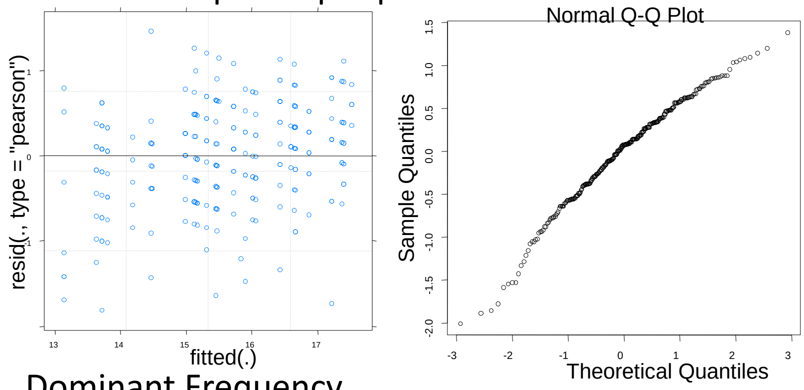

Dominant Frequency

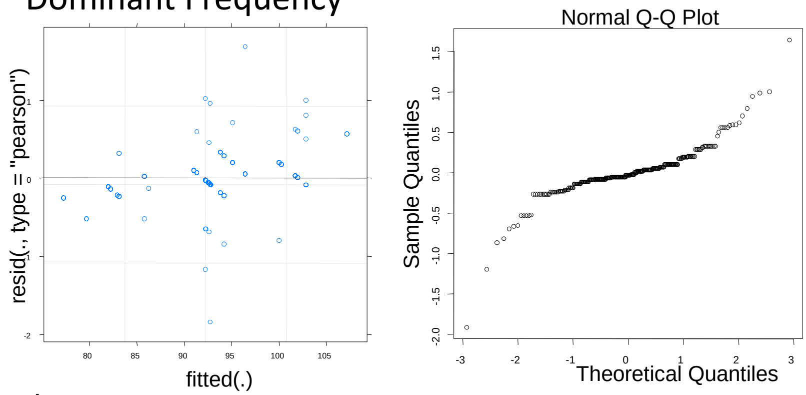

Chinavia impicticrnis MS-2

Pulse duration

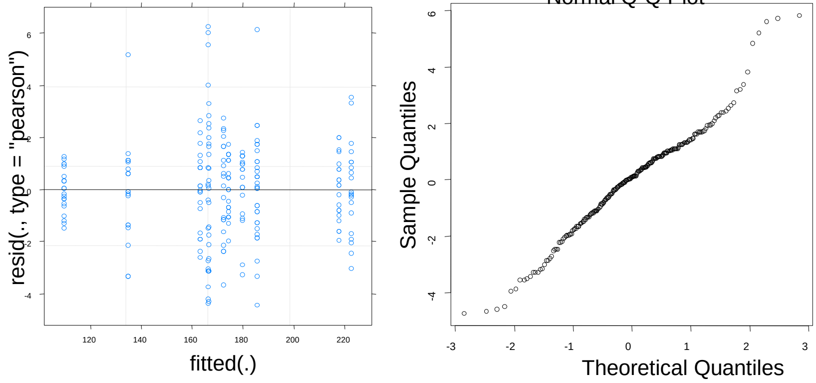

Pulse train repetition time

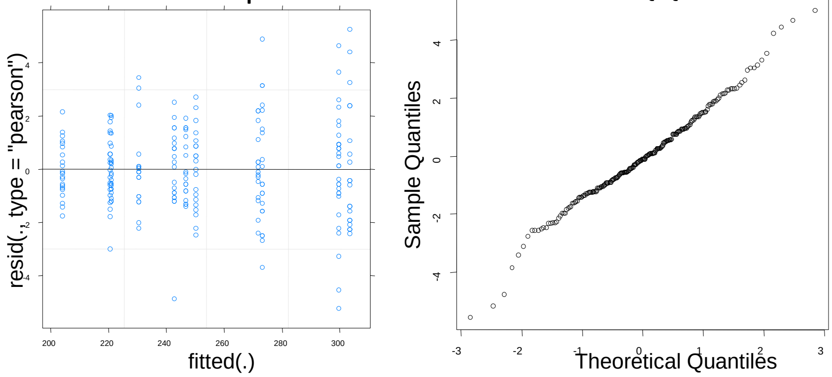

Dominant Frequency

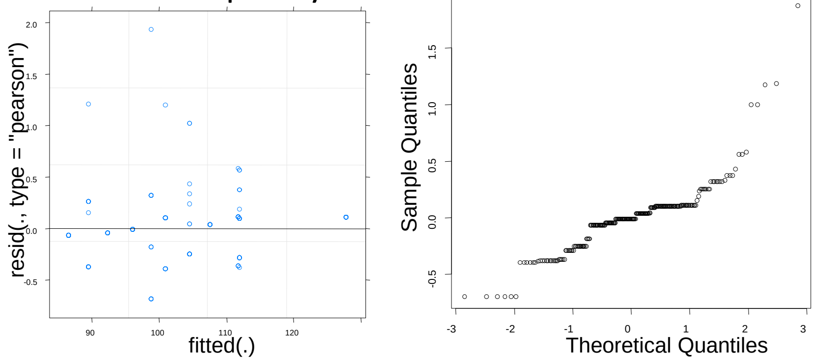

Supplement: Supplementary file 1 [file insects-12-00177-s001.zip › insects-1062341 Supplementary Material/insects-1062341 Figure S3.pdf]
