# Supplementary material for: Inhibitory Copulation Effect of Vibrational Rival Female Signals of Three Stink Bug Species as a Tool for Mating Disruption
Source: Insects. 2021 Feb 18;12(2):177. doi: 10.3390/insects12020177 (PMC7923018; doi:10.3390/insects12020177)

Supplementary material Figure S2

Inhibitory copulation effect of vibrational rival female signals of three stink bug species as a tool for mating disruption

Aline Moreira Dias, Miguel Borges, Maria Carolina Blassioli Moraes, Matheus Lorran Figueira Coelho, Andrej Čokl, Raul Alberto Laumann

Figure S2. General setup of the play back experiments.

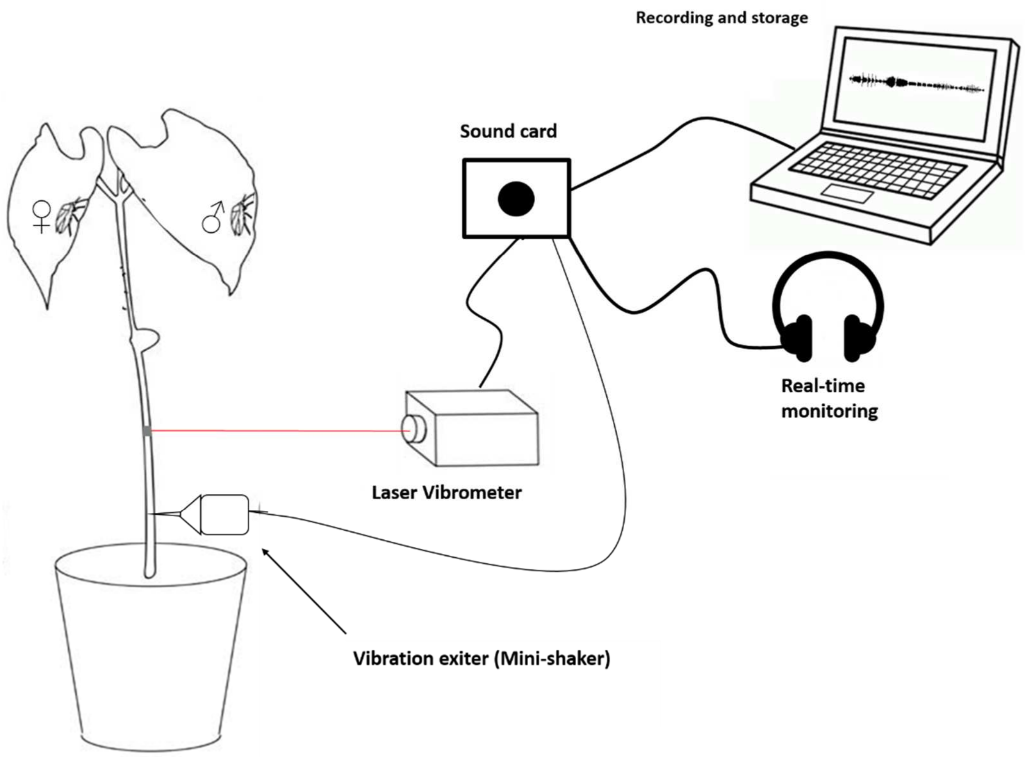

Supplement: Supplementary file 1 [file insects-12-00177-s001.zip › insects-1062341 Supplementary Material/insects-1062341 Figure S2.pdf]
